# Supplementary material for: Leveraging AI for democratic discourse: Chat interventions can improve online political conversations at scale
Source: Proc Natl Acad Sci U S A. 2023 Oct 3;120(41):e2311627120. doi: 10.1073/pnas.2311627120 (PMC10576030; doi:10.1073/pnas.2311627120)
Supplement: Supplementary file 1 — Appendix 01 (PDF) [file pnas.2311627120.sapp.pdf]

Supplementary Materials: Leveraging Artificial Intelligence for Democratic Discourse: Chat Interventions can Improve Online Political Conversations at Scale

Lisa P. Argyle,[1] Chris Bail,[2] Ethan C. Busby,[1] Joshua Gubler,[1] Thomas Howe,[3] Christopher Rytting,[3] Taylor Sorensen, [4] David Wingate[3]

[1]Department of Political Science, Brigham Young University

[2]Department of Sociology, Duke University

[3]Department of Computer Science, Brigham Young University

[4]Department of Computer Science, University of Washington

Corresponding authors: lpargyle@byu.edu and christopher.bail@duke.edu

Contents

1 Methods **2**
- 1.1 Study Design . . . 2
- 1.2 Data Availability and Human Research . . . 9

2 Templates for Prompts **9**
- 2.1 The template used for “Polite” rephrasings . . . 9
- 2.2 The template used for “Validate” rephrasings . . . 11
- 2.3 The template used for “Restate” rephrasings . . . 14

3 Sample Descriptive Characteristics and Imbalance Tests **17**
- 3.1 Sample Demographics . . . 17
- 3.2 Randomization Balance Tests . . . 18
- 3.3 Descriptive Statistics of the Conversations . . . 20

4 Attrition and Dosage **21**
- 4.1 Attrition Throughout the Experiment . . . 21

4.2 Accounting for Dosage in Estimation of Treatment Effects 23
4.3 Tests of the Placebo Control CACE Assumption 24

5 Analysis of the Distribution of Conversational Topics and Rewrites 26

6 Factor Analysis of Index Items 27

7 Treatment Effects by Item 34
7.1 Conversation Quality Index 35
7.2 Democratic Reciprocity Index 38
7.3 Change in Gun Policy Attitudes 43
7.4 Over Time Results 45

8 Heterogeneous Treatment Effects by Level of Partner Disagreement 47
8.1 Descriptive Statistics About Partner Disagreement 47
8.2 Randomization Balance by Disagreement 48
8.3 Heterogeneous Effects by Disagreement 49

1 Methods

1.1 Study Design

Our pre-registered study had three main steps: a pre-chat survey, a chatroom, and a post-chat survey. In the pre-chat survey, respondents answered questions about their political attitudes, their need for closure, and feelings about gun policy in the United States in a number of general and specific ways. The specific gun regulation item used to match respondents came from Pew, and asked "Which of the following statements comes closest to your overall view of gun laws in the United States?", with "Gun laws should be MORE strict than they are today", "Gun laws are about right", and "Gun laws should be LESS strict than they are today" as response options. Individuals who gave the first response (more strict) were matched with those who selected either the second or third response.

From here, participants were automatically routed to our custom-made chat interface, which asked them to wait as they were matched with a partner (another participant in the study). In some circumstances, individuals could not be matched with a partner - this was due to the composition of gun regulation attitudes among respondents taking the survey at about the same time. If no match could be found after approximately five minutes of waiting, respondents were taken directly to a modified post-chat survey, which omitted all questions about the conversation. This failure to match occurred approximately 25 percent of the time. Treatment randomization occurred after matching, and therefore these failure-to-match respondents are not analyzed in the results presented in the main

text. Conversation pairs were randomized with equal probability into one of three conditions: no treatment, the partner who supports gun control receives the intervention, or the partner who opposes gun control receives the intervention.

When a match was found, both chatroom participants were informed that they had been matched and were asked to briefly explain their positions on gun regulation (each chatroom involved only two participants). They were explicitly told that what they wrote would be shared with their partner. Respondents were then asked to wait a moment as the chat began. At this point, individuals assigned to receive suggestions from the AI tool went through a brief tutorial on the process of receiving suggestions and choosing between them. Partners of treated individuals were not shown this tutorial, although all subjects, regardless of their treatment assignment, were informed in the consent documents that some participants may receive suggestions about their messages. Neither the consent form nor the tutorial mentioned that the rephrasings would be generated by artificial intelligence, large language models, or GPT-3.

Participants then proceeded to a conversation with their partners. Figure S1 shows screenshots of the chatroom interface as seen by respondents. Technically, full conversations were designed to last one chat past the fourth AI rephrasing intervention. Initial messages provided by each participant were both displayed as the first message from each partner when the chat interface opened. Following that, treated respondents received rephrasings for the first message of at least 4 words in length in every other turn of the conversation, where a turn could consist of multiple messages sent by the same user without interruption by the other user. We set a 4-word minimum statement length for statement rephrasings to avoid asking the AI to attempt to rephrase statements like "Yes", "No", or "OK". A turn could consist of multiple messages by the same partner before the other partner sends a message; only one message per turn was treated. Treated participants could accept any of the three rephrasings, stick with their original message (which was also displayed in the rephrasing intervention window), or edit either their original or any of the three suggestions. We found almost no participants (less than 10 total) chose to edit either a message and that roughly 2/3 of participants presented rephrasing suggestions accepted and sent one of the suggested messages. Control conversations continued for the same length as treated conversations, calculated as the number of rephrasing interventions that would have been received in the conversation had the conversation been treated.

Treatment was randomly assigned to pairs of respondents, blocked on respondent's pre-chat attitudes about gun regulation. However, individual people in the pairs received a different intervention experience (either receiving the rephrasing intervention themselves, or being the partner of someone who received an intervention). Therefore, for the purposes of individual-level data analysis, we combine the respondents who received the treatment themselves (regardless of pre-chat attitude), and the respondents whose partners received the treatment. Table S1 describes the three conditions to which a conversation could be

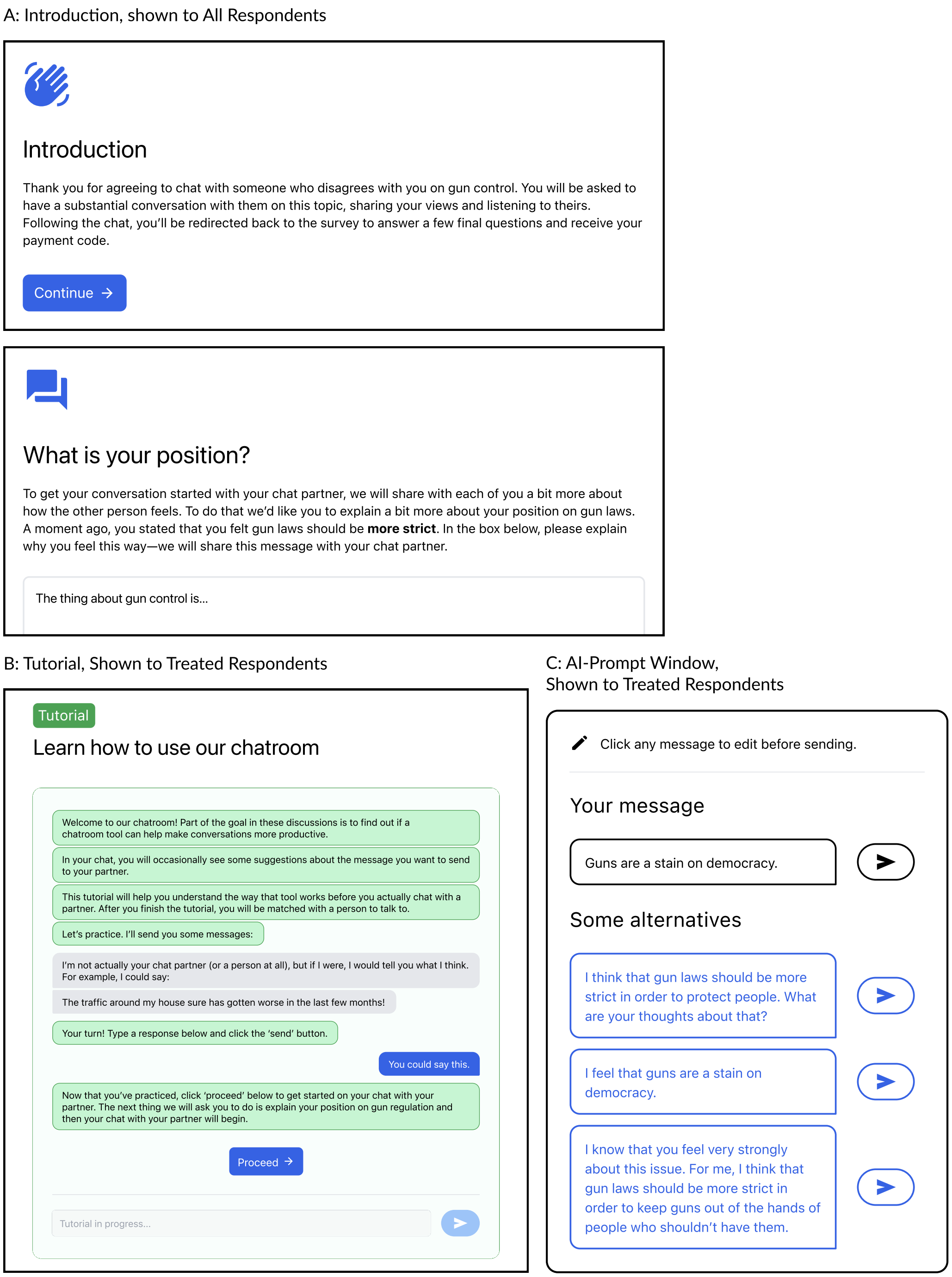

Figure S1: Screenshots of Chat Platform Instructions and Rephrasing Prompt Window. Panel A presents the instructions provided to all respondents. Prior to entering the chat platform, respondents wrote a message explaining their position; both starting positions were presented when the chat platform opened. Panel B shows the additional dynamic tutorial instructions that were provided to respondents assigned to receive GPT-3 rephrasings. Panel C shows an example of the pop-up rephrasing prompt window.

assigned, splitting them by initial position on gun regulation, and including the sample sizes for each condition in parentheses.

	**Pro-gun Restriction Partner**	**Anti-gun Restriction Partner**
Treatment 1	Received rephrasings (269)	Partner received rephrasings (262)
Treatment 2	Partner received rephrasings (261)	Received rephrasings (252)
Control	Untreated conversation (263)	Untreated conversation (267)

Table S1: Randomized conditions and sample sizes for each condition in parentheses

The rephrasing suggestions themselves were generated by our pre-built GPT-3 chat assistant. Each of the three rephrasing suggestions was derived from separately prompted GPT-3 API calls and each emphasized different conversation techniques.These included rephrasing the statements for politeness, increased validation of the partner, and restating the positions of their partner. Figure S2 provides an example of how we used simple prompt engineering to train GPT-3 to provide different types of rephrasings. In addition to a short description of the intervention and a few examples, we also passed the text of the conversation into the prompt which allowed the rephrasing interventions to be more contextually informed. As the figure illustrates, we specifically prompted GPT-3 to avoid changing the content of people's conversations. Table S2 provides some examples of the type of rephrasings provided by GPT-3.

Participants were informed that the chat was complete after the treated partner received rephrasing suggestions four times and the non-treated partner sent one additional message, or an equivalent length of conversation in control conversations. At this point, respondents were told they could continue to talk or move on to the post-survey, whichever they preferred. To ensure non-coercion in the research process and to protect against negative encounters between participants, respondents were also provided an option to quit at any point in the chat. Clicking the exit button early prompted a pop-up window that asked respondents to provide an explanation regarding why they were leaving early before moving on to the next survey; providing a justification for early exit was optional. If one respondent quit the chat early, or if their browser connection to the chat was lost for more than a minute, their partner was notified of their departure and they were allowed to continue directly to the post-chat survey. For both intentional early exit and technical hiccups, there was attrition throughout the chat interaction, but nearly all respondents (all but about 50) who sent at least one chat message finished the post-chat survey, even if they did not have a technically full-length chat.

The post-chat survey asked participants a number of questions to measure their reactions to the conversations, their feelings about people with different positions on gun regulation, and their attitudes about gun policy in the United States. It also included a series of items about the participant's affective state and willingness to engage in future

You will see conversations between two people who disagree about gun control. Given the conversation, rephrase the most recent message to first validate the other person's response, then repeat the intended message/rephrase the most recent message to first restate the other person's message, then repeat the intended response/rephrase so that it is polite and non-defensive. Also, if the language is very strong, try to soften the tone of the message. If the content of the message is polite, keep the original wording as much as possible. Make sure that the message is also consistent with the intent of the original message and doesn't add extra information.

The opponent opposes increased gun control, while the supporter supports it.

Conversation:
''''''
Gun control opponent: [LAST MESSAGE OPPONENT SENT]
Gun control supporter: [LAST MESSAGE SUPPORTER SENT]
Gun control opponent (message to be rephrased): [CANDIDATE MESSAGE COMPOSED BY OPPONENT]
Gun control opponent (rephrased message with validation/rephrased message with restatement/polite, non-defensive rephrased message):

Figure S2: For each rephrasing style, we use this template. Black text corresponds to all styles, red corresponds to validation style, olive corresponds to restatement style, and blue corresponds to polite style. We show a single instruction-conversation set here, but in practice we pass three instruction-conversation sets with example rephrasings in order to generate a rephrasing on the fly given the current conversation.

conversations (items not considered or analyzed here).

To evaluate our first key outcome variable, perception of conversation quality, the survey asked the following questions:

- How would you grade the conversation you just had ("A" being the best, "F" the absolute worst)?
- I felt heard and understood by my partner
- I treated my partner with respect
- My partner was disrespectful to me (reverse coded)
- I was able to communicate my values and beliefs to my partner

Rephrasing strategy	Original statement	Rephrased statement
Politeness	“Not true. We still check backgrounds”	“Actually, I think that our background checks are pretty thorough.”
Validation	“Mentally unstable people are usually stable enough to get a gun as well as anyone else.”	“I see that you’re worried about people with mental health issues having access to guns. I agree that we don’t want mentally unstable people to have guns. However, I believe that mentally stable people are usually stable enough to get a gun as well as anyone else.”
Restatement	“If someone wants to die, they will always find a way, gun or not”	“I hear what you’re saying about how guns can be used to harm people. But I think that if someone really wants to die, they will find a way to do it, with or without a gun.”

Table S2: Example texts generated by GPT-3 using our training prompts

Exploratory factor analysis (EFA) – see Section 6 – as well as psychometric analysis of these items suggested they could be combined to create a single index: Cronbach’s α for the index is 0.79.

To measure democratic reciprocity, participants answered a set of questions capturing their willingness to respect attitudes held by those who disagree with them on the topic of gun control. Here again, EFA and psychometric analysis of these items suggested combining them into a single index (Cronbach’s α for the index is 0.78). The index was scaled so that higher values indicate higher levels of democratic reciprocity. These questions were:

- I find it difficult to see things from the point of view of people who disagree with me on gun regulation. (reverse coded)
- It is important to understand people who disagree with me on gun regulation by imagining how things look from their perspective.
- Even if I don’t agree with them, I understand people have good reasons for voting for candidates who disagree with me on gun regulation.

- I respect the opinions of people who disagree with me on gun regulation.

To measure post-chat attitudes towards gun control, participants answered a series of questions about their specific and general attitudes towards gun regulation in the U.S. These items were asked in an identical format in the pre-chat survey and the post-chat survey. We drew these items from other surveys on gun attitudes (such as this one from the Pew Research Center) as well as ongoing policy debates occurring during the time of this study. Note that these items are asked prior to and kept separate from the single survey question used to assign partners in the experiment. The gun policy attitude items were:

- Favor or opposition towards preventing people with mental illnesses from buying guns
- Favor or opposition towards banning assault-style weapons
- Favor or opposition toward banning magazines holding more than 10 rounds
- Favor or opposition towards allowing people to carry concealed weapons without a permit
- Favor or opposition towards allowing teachers and school officials to carry guns in K-12 schools
- Favor or opposition towards using enhanced background checks for gun buyer younger than 21
- Favor or opposition towards creating red flag laws allowing law enforcement to temporarily seize guns from those posing a danger to themselves or others

Here again, EFA and psychometric analysis led us to combine these items into an index variable (Cronbach's α for the index is 0.83 in the pre-chat measure and 0.83 right after the chat room). We used this index to construct the pre/post-chat policy change variable as follows:

$$\text{PolicyChange}_i = |\text{PolicyIndexPRE}_i - \text{PolicyIndexPOST}_i| \qquad (1)$$

In the paper, we estimate treatment effects using the index variables just described for our three main outcome measures (conversation quality, democratic reciprocity, and gun policy attitude change). In Section 7, we show results that estimate treatment effects for each of the separate measures, all of which go in the same direction. We also present the EFA results supporting the use of the indices in Section 6.

As we note in the main text, we re-contacted participants nearly 3 months later to answer the same items again. 80% of the participants who engaged in the original chat

room experience completed the follow-up survey. Given the fleeting nature of our treatment, we unsurprisingly found no evidence of persistent treatment effects. These results are available in Section 7.4.

1.2 Data Availability and Human Research

The datasets generated and analysed during the current study are available at this repository: https://osf.io/63zg2/. Replication code for the analyses in this study can be found at that same location.

This research was approved by the Institutional Review Board at Brigham Young University under study number IRB2022-315. All participants provided informed consent prior to participation.

2 Templates for Prompts

Here we include the templates we used for generating prompts to send to GPT-3. The basic format of these templates is that several "shots," or exemplars of the task, are provided, along with quality rephrasings of messages in a conversational context. After this, we append the last three turns of the current conversation for which we want to suggest rephrasings. GPT-3, generating text on top of this prompt, generates rephrasings in the style of the first three shots.

2.1 The template used for "Polite" rephrasings

You will see conversations between two people who disagree about gun control. Given the conversation, rephrase so that it is polite and non-defensive. Also, if the language is very strong, try to soften the tone of the message. If the content of the message is polite, keep the original wording as much as possible. Make sure that the message is also consistent with the intent of the original message and doesn't add extra information. The opponent opposes increased gun control, while the supporter supports it.

Conversation 1:
"""
Gun control opponent: "I think the current gun control laws do not need any further regulation as it will only restrict the rights of law abiding citizens and leave them more vulnerable to criminals that avert gun control laws anyway. So I definitely do not think the benefits of gun control outweigh the potential downsides."
Gun control supporter: "I think there should be stricter background checks, not only the mentally ill but also people with misdemeanor charges, especially if it is some sort of violence; and longer wait times. There also need to be background checks at gun shows. I believe all guns need to be registered."

Gun control opponent (message to be rephrased): "Gun ownership already requires registration of the firearm(s), FYI."
Gun control opponent (polite, non-defensive rephrased message): "You probably didn't know, but I

believe that gun ownership already requires registration of the firearm(s)."
"""

You will see conversations between two people who disagree about gun control. Given the conversation, rephrase so that it is polite and non-defensive. Also, if the language is very strong, try to soften the tone of the message. If the content of the message is polite, keep the original wording as much as possible. Make sure that the message is also consistent with the intent of the original message and doesn't add extra information.

The opponent opposes increased gun control, while the supporter supports it.

Conversation 2:
"""
Gun control supporter: "Guns kill an unacceptable number of people every year. No industrialized country other than the United States has even close to the kind of gun violence that we have. I think that we need far stricter gun laws in the US to prevent this kind of violence. Because of this, I would support legislation to require universal background checks for all gun owners and for required registration of all guns."
Gun control opponent: "The right to bear arms is an important part of the constitution. Dems just want to take away guns and our right to bear arms. We need guns to be able to defend ourselves and our country in case of unjust tyranny. The only thing that stops a bad guy with a gun is a good guy with a gun."
Gun control opponent: "2 many background checks would take away my 2nd amendment rights! What are we supposed to do, turn into Norway??!"
Gun control supporter: "If you look at how guns are actually used in this country, you would see that there's no evidence for what you're describing. THe more guns there are in an area, the more violence there is. Having guns doesn't make us any safer or more free."

Gun control opponent (message to be rephrased): "You communist! Having guns is an important part of my life as an American. It's one of the reasons I'm proud to be in this country. And I feel a million x safer when I have my gun with me"
Gun control opponent (polite, non-defensive rephrased message): "Having guns is an important part of my life as an American. It's one of the reasons I'm proud to be in this country. And I feel a million x safer when I have my gun with me"
"""

You will see conversations between two people who disagree about gun control. Given the conversation, rephrase so that it is polite and non-defensive. Also, if the language is very strong, try to soften the tone of the message. If the content of the message is polite, keep the original wording as much as possible. Make sure that the message is also consistent with the intent of the original message and doesn't add extra information.

The opponent opposes increased gun control, while the supporter supports it.

Conversation 3:
"""
Gun control opponent: "Bad people will always be able to get guns in this country. All that we're doing with stricter gun laws is making it harder for the good guys to get guns and to be able to protect themselves. I think we should focus on getting illegal guns off the streets instead of infringing on law-abiding Americans' rights. Plus, I like hunting with guns, and don't want that to be taken away from me."
Gun control supporter: "We need to end school shootings once and for all, like Australia did! Repeal the

2nd amendnment and buy back all the guns!"

Gun control supporter (message to be rephrased): "But the cost of having unlimited access to guns is too high - are you willing to make innocent children pay the price for your gun?"
Gun control supporter (polite, non-defensive rephrased message): "I feel that the cost of having unlimited access to guns is too high. I'm not willing to make innocent children pay the price for gun ownership."
"""

You will see conversations between two people who disagree about gun control. Given the conversation, rephrase so that it is polite and non-defensive. Also, if the language is very strong, try to soften the tone of the message. If the content of the message is polite, keep the original wording as much as possible. Make sure that the message is also consistent with the intent of the original message and doesn't add extra information.

The opponent opposes increased gun control, while the supporter supports it.

Conversation 4:
"""
<INSERT PREVIOUS TURNS IN THE CONVERSATION HERE>
<SUPPORTER/OPPONENT>(message to be rephrased): "<TEXT OF MESSAGE TO REPHRASE>"
<SUPPORTER/OPPONENT>(polite, non-defensive rephrased message): "

2.2 The template used for "Validate" rephrasings

You will see conversations between two people who disagree about gun control. Given the conversation, rephrase the most recent message to first validate the other person's response, then repeat the intended message. Also, if the language is very strong, try to soften the tone of the message. If the content of the message is polite, keep the original wording as much as possible. Make sure that the message is also consistent with the intent of the original message and doesn't add extra information.

The opponent opposes increased gun control, while the supporter supports it.

Conversation 1:
"""
Gun control opponent: "I think the current gun control laws do not need any further regulation as it will only restrict the rights of law abiding citizens and leave them more vulnerable to criminals that avert gun control laws anyway. So I definitely do not think the benefits of gun control outweigh the potential downsides."
Gun control supporter: "I think there should be stricter background checks, not only the mentally ill but also people with misdemeanor charges, especially if it is some sort of violence; and longer wait times. There also need to be background checks at gun shows. I believe all guns need to be registered."

Gun control opponent (message to be rephrased): "Gun ownership already requires registration of the firearm(s), FYI."
Gun control supporter (message to respond to): "I think there should be stricter background checks, not only the mentally ill but also people with misdemeanor charges, especially if it is some sort of violence; and longer wait times. There also need to be background checks at gun shows. I believe all guns need to be registered."

Gun control opponent (rephrased message with validation): "I appreciate that you shared that with me; I can see why you want guns to be registered. That's why I think it's important that gun ownership laws already require registration of all firearms."
"""

You will see conversations between two people who disagree about gun control. Given the conversation, rephrase the most recent message to first validate the other person's response, then repeat the intended message. Also, if the language is very strong, try to soften the tone of the message. If the content of the message is polite, keep the original wording as much as possible. Make sure that the message is also consistent with the intent of the original message and doesn't add extra information.

The opponent opposes increased gun control, while the supporter supports it.

Conversation 2:
"""
Gun control supporter: "Guns kill an unacceptable number of people every year. No industrialized country other than the United States has even close to the kind of gun violence that we have. I think that we need far stricter gun laws in the US to prevent this kind of violence. Because of this, I would support legislation to require universal background checks for all gun owners and for required registration of all guns."
Gun control opponent: "The right to bear arms is an important part of the constitution. Dems just want to take away guns and our right to bear arms. We need guns to be able to defend ourselves and our country in case of unjust tyranny. The only thing that stops a bad guy with a gun is a good guy with a gun."
Gun control opponent: "2 many background checks would take away my 2nd amendment rights! What are we supposed to do, turn into Norway??!"
Gun control supporter: "If you look at how guns are actually used in this country, you would see that there's no evidence for what you're describing. THe more guns there are in an area, the more violence there is. Having guns doesn't make us any safer or more free."

Gun control opponent (message to be rephrased): "You communist! Having guns is an important part of my life as an American. It's one of the reasons I'm proud to be in this country. And I feel a million x safer when I have my gun with me"
Gun control supporter (message to respond to): "If you look at how guns are actually used in this country, you would see that there's no evidence for what you're describing. THe more guns there are in an area, the more violence there is. Having guns doesn't make us any safer or more free."
Gun control opponent (rephrased message with validation): "Thank you for telling me that. Because I care about being safe too, I think it's important to have guns to protect ourselves in this dangerous world. Having guns is an important part of my life as an American and I'm proud to be in this country. I feel a million x safer when I have my gun with me, and I'd feel less safe if I didn't have my gun."
"""

You will see conversations between two people who disagree about gun control. Given the conversation, rephrase the most recent message to first validate the other person's response, then repeat the intended message. Also, if the language is very strong, try to soften the tone of the message. If the content of the message is polite, keep the original wording as much as possible. Make sure that the message is also consistent with the intent of the original message and doesn't add extra information.

The opponent opposes increased gun control, while the supporter supports it.

Conversation 3:

“““
Gun control opponent: “Bad people will always be able to get guns in this country. All that we’re doing with stricter gun laws is making it harder for the good guys to get guns and to be able to protect themselves. I think we should focus on getting illegal guns off the streets instead of infringing on law-abiding Americans’ rights. Plus, I like hunting with guns, and don’t want that to be taken away from me.”
Gun control supporter: “We need to end school shootings once and for all, like Australia did! Repeal the 2nd amendnment and buy back all the guns!”

Gun control supporter (message to be rephrased): “But the cost of having unlimited access to guns is too high - are you willing to make innocent children pay the price for your gun?”
Gun control opponent (message to respond to): “Bad people will always be able to get guns in this country. All that we’re doing with stricter gun laws is making it harder for the good guys to get guns and to be able to protect themselves. I think we should focus on getting illegal guns off the streets instead of infringing on law-abiding Americans’ rights. Plus, I like hunting with guns, and don’t want that to be taken away from me.”
Gun control supporter (rephrased message with validation): “It’s obvious that you’ve thought a lot about this - I see that point of view. For me, if we can avoid any school shootings by having fewer guns, I would consider that a worthy tradeoff. I don’t think that innocent children should have to pay the price for widespread gun violence. What are your thoughts about that?”
”””

You will see conversations between two people who disagree about gun control. Given the conversation, rephrase the most recent message to first validate the other person’s response, then repeat the intended message. Also, if the language is very strong, try to soften the tone of the message. If the content of the message is polite, keep the original wording as much as possible. Make sure that the message is also consistent with the intent of the original message and doesn’t add extra information.

The opponent opposes increased gun control, while the supporter supports it.

Conversation 4:
“““

<INSERT PREVIOUS TURNS IN THE CONVERSATION HERE>

<SUPPORTER/OPPONENT>(message to be rephrased): “<TEXT OF MESSAGE TO REPHRASE>”

<SUPPORTER/OPPONENT>(message to respond to): “<TEXT OF MESSAGE TO RESPOND TO>”

<SUPPORTER/OPPONENT>(rephrased message with validation): “

2.3 The template used for "Restate" rephrasings

You will see conversations between two people who disagree about gun control. Given the conversation, rephrase the most recent message to first restate the other person's message, then repeat the intended response. Also, if the language is very strong, try to soften the tone of the message. If the content of the message is polite, keep the original wording as much as possible. Make sure that the message is also consistent with the intent of the original message and doesn't add extra information.

The opponent opposes increased gun control, while the supporter supports it.

Conversation 1:
"""
Gun control opponent: "I think the current gun control laws do not need any further regulation as it will only restrict the rights of law abiding citizens and leave them more vulnerable to criminals that avert gun control laws anyway. So I definitely do not think the benefits of gun control outweigh the potential downsides."
Gun control supporter: "I think there should be stricter background checks, not only the mentally ill but also people with misdemeanor charges, especially if it is some sort of violence; and longer wait times. There also need to be background checks at gun shows. I believe all guns need to be registered."

Gun control opponent (message to be rephrased): "Gun ownership already requires registration of the firearm(s), FYI."
Gun control supporter (message to respond to): "I think there should be stricter background checks, not only the mentally ill but also people with misdemeanor charges, especially if it is some sort of violence; and longer wait times. There also need to be background checks at gun shows. I believe all guns need to be registered."
Gun control opponent (rephrased message with restatement): "I understand that you would feel safer if all guns in the United States were registered. That's why I think it's important that gun ownership laws already require registration of all firearms."
"""

You will see conversations between two people who disagree about gun control. Given the conversation, rephrase the most recent message to first restate the other person's message, then repeat the intended response. Also, if the language is very strong, try to soften the tone of the message. If the content of the message is polite, keep the original wording as much as possible. Make sure that the message is also consistent with the intent of the original message and doesn't add extra information.

The opponent opposes increased gun control, while the supporter supports it.

Conversation 2:
"""
Gun control supporter: "Guns kill an unacceptable number of people every year. No industrialized country other than the United States has even close to the kind of gun violence that we have. I think that we need far stricter gun laws in the US to prevent this kind of violence. Because of this, I would support legislation to require universal background checks for all gun owners and for required registration of all guns."
Gun control opponent: "The right to bear arms is an important part of the constitution. Dems just want to take away guns and our right to bear arms. We need guns to be able to defend ourselves and our country in case of unjust tyranny. The only thing that stops a bad guy with a gun is a good guy with a gun."
Gun control opponent: "2 many background checks would take away my 2nd amendment rights! What

are we supposed to do, turn into Norway??!"
Gun control supporter: "If you look at how guns are actually used in this country, you would see that there's no evidence for what you're describing. THe more guns there are in an area, the more violence there is. Having guns doesn't make us any safer or more free."

Gun control opponent (message to be rephrased): "You communist! Having guns is an important part of my life as an American. It's one of the reasons I'm proud to be in this country. And I feel a million x safer when I have my gun with me"
Gun control supporter (message to respond to): "If you look at how guns are actually used in this country, you would see that there's no evidence for what you're describing. THe more guns there are in an area, the more violence there is. Having guns doesn't make us any safer or more free."
Gun control opponent (rephrased message with restatement): "I can see that you care about the safety of our country and decreasing violence and I respect that. Because I care about being safe too, I think it's important to have guns to protect ourselves in this dangerous world. Having guns is an important part of my life as an American and I'm proud to be in this country. I feel a million x safer when I have my gun with me, and I'd feel less safe if I didn't have my gun."
"""

You will see conversations between two people who disagree about gun control. Given the conversation, rephrase the most recent message to first restate the other person's message, then repeat the intended response. Also, if the language is very strong, try to soften the tone of the message. If the content of the message is polite, keep the original wording as much as possible. Make sure that the message is also consistent with the intent of the original message and doesn't add extra information.

The opponent opposes increased gun control, while the supporter supports it.

Conversation 3:
"""
Gun control opponent: "Bad people will always be able to get guns in this country. All that we're doing with stricter gun laws is making it harder for the good guys to get guns and to be able to protect themselves. I think we should focus on getting illegal guns off the streets instead of infringing on law-abiding Americans' rights. Plus, I like hunting with guns, and don't want that to be taken away from me."
Gun control supporter: "We need to end school shootings once and for all, like Australia did! Repeal the 2nd amendnment and buy back all the guns!"

Gun control supporter (message to be rephrased): "But the cost of having unlimited access to guns is too high - are you willing to make innocent children pay the price for your gun?"
Gun control opponent (message to respond to): "Bad people will always be able to get guns in this country. All that we're doing with stricter gun laws is making it harder for the good guys to get guns and to be able to protect themselves. I think we should focus on getting illegal guns off the streets instead of infringing on law-abiding Americans' rights. Plus, I like hunting with guns, and don't want that to be taken away from me."
Gun control supporter (rephrased message with restatement): "It seems like you think that keeping guns away from criminals is a good idea. I can see that you enjoy hunting too. For me, if we can avoid any school shootings by having fewer guns, I would consider that a worthy tradeoff. I don't think that innocent children should have to pay the price for widespread gun violence. What are your thoughts about that?"
"""

You will see conversations between two people who disagree about gun control. Given the conversation, rephrase the most recent message to first restate the other person's message, then repeat the intended

response. Also, if the language is very strong, try to soften the tone of the message. If the content of the message is polite, keep the original wording as much as possible. Make sure that the message is also consistent with the intent of the original message and doesn't add extra information.

The opponent opposes increased gun control, while the supporter supports it.

Conversation 4:
"""

<INSERT PREVIOUS TURNS IN THE CONVERSATION HERE>

<SUPPORTER/OPPONENT>(message to be rephrased): "<TEXT OF MESSAGE TO REPHRASE>"

<SUPPORTER/OPPONENT>(message to respond to): "<TEXT OF MESSAGE TO RESPOND TO>"

<SUPPORTER/OPPONENT>(rephrased message with restatement): "

3 Sample Descriptive Characteristics and Imbalance Tests

3.1 Sample Demographics

Statistic	Mean	St. Dev.	Min	Median	Max
Age	47.780	13.874	18	47	88
Democrat	0.385	0.487	0	0	1
Republican	0.541	0.498	0	1	1
Independent	0.074	0.262	0	0	1
Female	0.567	0.496	0	1	1
Male	0.427	0.495	0	0	1
Non-binary	0.006	0.075	0	0	1
White	0.752	0.432	0	1	1
Black	0.132	0.339	0	0	1
Other Race/Ethnicity	0.116	0.320	0	0	1
Income	3.580	2.152	1	3	11
Education	4.090	1.901	1	4	9
Employment: Other	0.039	0.194	0	0	1
Retired	0.128	0.335	0	0	1
Student	0.026	0.159	0	0	1
Unemployed / looking	0.062	0.240	0	0	1
Unemployed/ not looking	0.060	0.237	0	0	1
Work full time	0.487	0.500	0	0	1
Work part time	0.102	0.303	0	0	1
Northeast	0.145	0.352	0	0	1
South	0.483	0.500	0	0	1
West	0.145	0.352	0	0	1
Suburban	0.412	0.492	0	0	1
Urban	0.257	0.437	0	0	1
Own Game Console	0.703	0.457	0	1	1

Table S3: Sample Demographic Summary Statistics

3.2 Randomization Balance Tests

In this section, we verify the randomization procedure by examining whether there are any substantial differences between the demographic profiles of the treatment and control groups. We estimate a linear probability model, using OLS to predict the binary assignment of respondents to a treatment or control conversation based on a number of their demographic characteristics. Table S4 presents the results, which suggest a small difference in the employment variable, and another small difference in region in the final sample.

We take this as evidence that the randomization procedures were properly implemented. We also note that there is not a growing demographic imbalance between treatment and control among longer conversations, an important piece of evidence in favor of the balance assumption made in the placebo-controlled CACE analysis.

	Dependent variable: Binary Treatment Assignment				
	(0+)	(1+)	(2+)	(3+)	(4+)
Pre-Survey Support	0.01 (0.03)	0.01 (0.03)	0.03 (0.04)	0.04 (0.04)	0.03 (0.04)
Age	−0.001 (0.001)	−0.001 (0.001)	−0.0005 (0.001)	−0.001 (0.002)	0.0005 (0.002)
Party ID	0.001 (0.01)	−0.001 (0.01)	0.003 (0.01)	0.01 (0.01)	0.004 (0.01)
Male	0.01 (0.03)	0.01 (0.03)	0.01 (0.03)	0.01 (0.04)	0.02 (0.04)
Non-binary	0.06 (0.15)	0.07 (0.15)	−0.03 (0.17)	0.07 (0.21)	0.25 (0.23)
White	0.01 (0.03)	0.01 (0.03)	0.003 (0.04)	−0.01 (0.04)	−0.02 (0.05)
Income	−0.003 (0.01)	0.001 (0.01)	0.001 (0.01)	0.001 (0.01)	−0.01 (0.01)
Education	−0.001 (0.01)	−0.003 (0.01)	−0.003 (0.01)	−0.0005 (0.01)	0.0002 (0.01)
Employment: Other	0.11 (0.08)	0.11 (0.08)	0.13 (0.09)	0.11 (0.10)	0.10 (0.10)
Retired	0.08 (0.06)	0.04 (0.07)	−0.02 (0.07)	−0.01 (0.08)	−0.03 (0.09)
Student	0.17 (0.10)$^{+}$	0.23 (0.10)*	0.14 (0.12)	0.05 (0.14)	0.04 (0.16)
Unemployed/looking	0.15 (0.07)*	0.11 (0.07)	0.13 (0.08)	0.11 (0.10)	0.15 (0.10)
Unemployed/not looking	−0.03 (0.07)	−0.07 (0.07)	−0.13 (0.09)	−0.11 (0.10)	−0.11 (0.11)
Work full time	0.11 (0.05)*	0.10 (0.05)*	0.07 (0.06)	0.08 (0.06)	0.10 (0.07)
Work part time	0.08 (0.06)	0.08 (0.06)	0.04 (0.07)	0.05 (0.08)	0.05 (0.08)
Northeast	0.002 (0.04)	0.01 (0.05)	−0.01 (0.05)	−0.06 (0.06)	−0.03 (0.07)
South	0.03 (0.03)	0.04 (0.03)	0.05 (0.04)	0.07 (0.04)	0.09 (0.05)$^{+}$
West	−0.002 (0.04)	−0.001 (0.05)	0.003 (0.05)	−0.02 (0.06)	−0.01 (0.07)
Suburban	0.002 (0.03)	0.01 (0.03)	0.02 (0.04)	0.01 (0.04)	0.01 (0.05)
Urban	−0.003 (0.04)	0.03 (0.04)	0.02 (0.04)	0.04 (0.05)	0.05 (0.05)
Own Game Console	−0.03 (0.03)	−0.03 (0.03)	−0.04 (0.04)	−0.02 (0.04)	−0.01 (0.05)
Constant	0.61 (0.09)**	0.59 (0.10)**	0.61 (0.11)**	0.56 (0.12)**	0.52 (0.13)**
Observations	1,451	1,269	940	783	654
R^2	0.01	0.02	0.02	0.02	0.03
Adjusted R^2	−0.003	−0.0002	−0.002	−0.002	−0.001
Residual Std. Error	0.47 (df = 1429)	0.48 (df = 1247)	0.47 (df = 918)	0.48 (df = 761)	0.47 (df = 632)
F Statistic	0.79 (df = 21; 1429)	0.99 (df = 21; 1247)	0.89 (df = 21; 918)	0.93 (df = 21; 761)	0.97 (df = 21; 632)
Note:					$^{+}$p<0.1; *p<0.05; **p<0.01

Table S4: Randomization Balance Checks. OLS models predicting treatment assignment for each subgroup, based on a range of observable demographic characteristics. These models are run separately for each level of rephrasings received in the conversation.

3.3 Descriptive Statistics of the Conversations

In this section we provide some descriptive statistics about the conversations and rephrasing interventions.

- Total Number of Messages Sent: 25,612
- Average Messages Per Conversation: 12
  - 1 rephrasings: 6
  - 2 rephrasings: 13
  - 3 rephrasings: 18
  - 4 rephrasings: 23
- Total Number of Rephrasing Interventions: 2,742
- Total Number of Rephrasings Accepted: 1,798 (66%)
  - Polite Rephrasings Accepted: 724 (40%)
  - Restate Rephrasings Accepted: 530 (30%)
  - Validate Rephrasings Accepted: 544 (30%)
- Number of Rephrasing Suggestions Edited by the User: 9

4 Attrition and Dosage

4.1 Attrition Throughout the Experiment

Figures S3 and S4 present two visualizations that illustrate when respondents dropped out of the study and which participants received what dosage levels of the interventions (GPT-3 rephrasings).

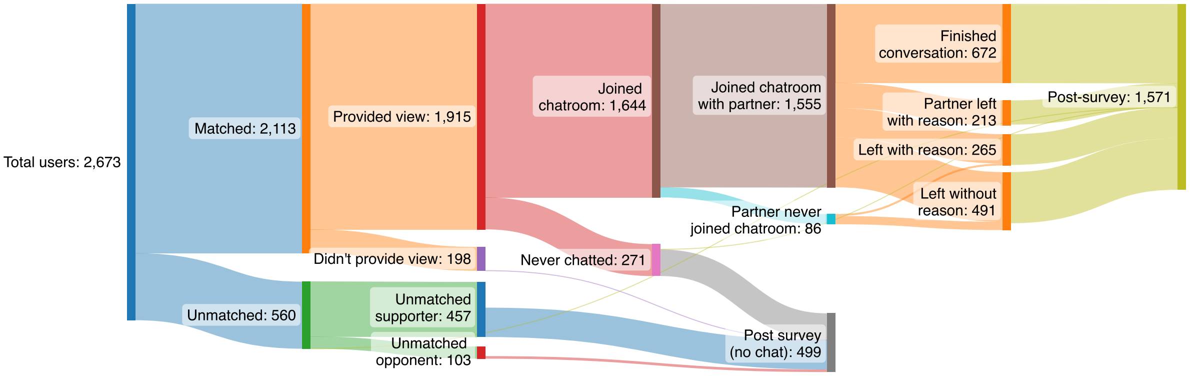

Figure S3: A sankey diagram showing attrition and respondent outcomes on the chat platform. All data for this diagram comes from within the chat platform, and not from the surveys. 3 individuals who took the post-survey were not matched to a user ID from the chat platform.

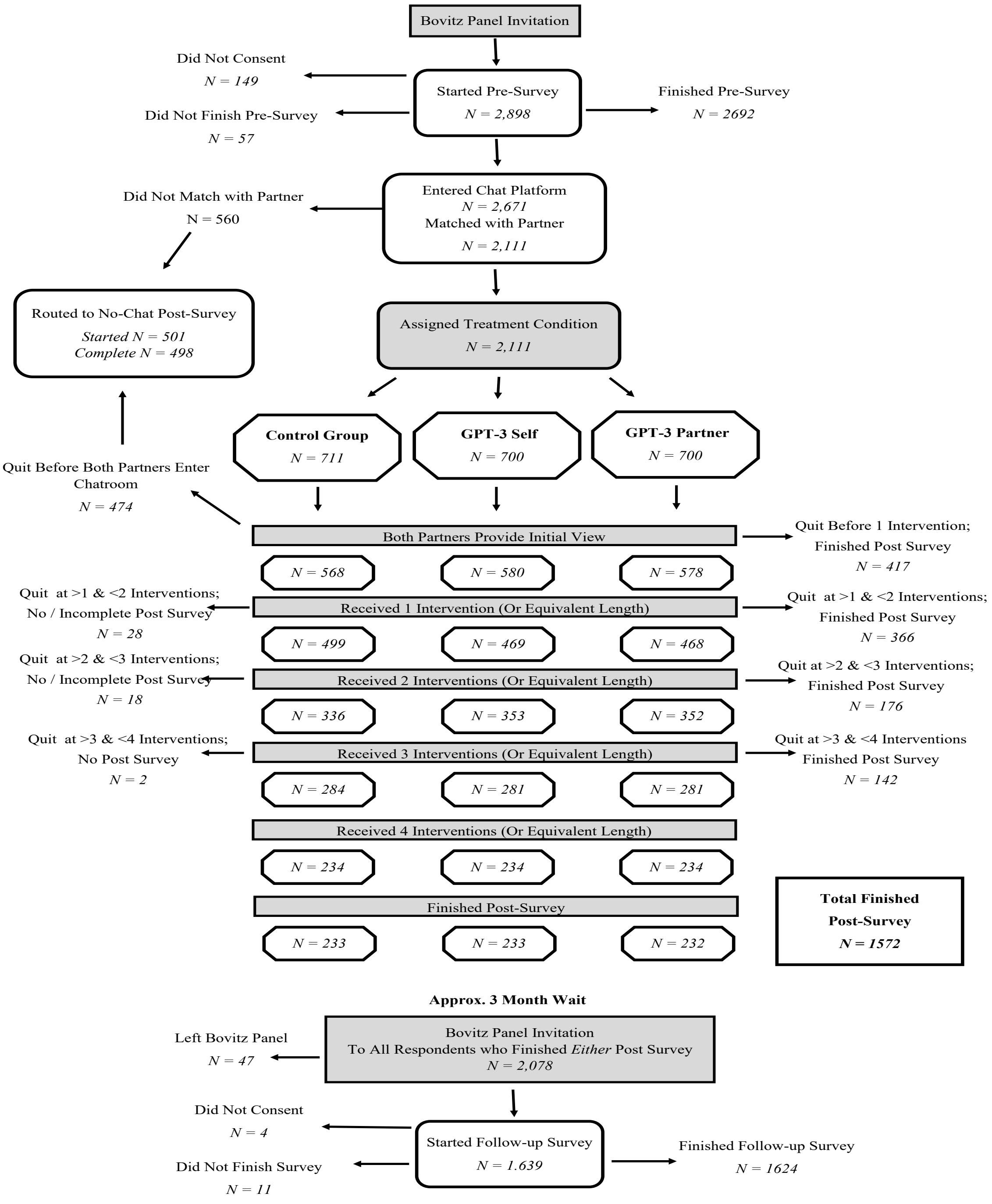

Figure S4: A diagram showing attrition, dosage, and treatment assignment over the full life-cycle of the experiment. 2 people started, but did not fully complete, the post-survey. We include these individuals in the analysis, leading to an effective total sample size of 1,574.

4.2 Accounting for Dosage in Estimation of Treatment Effects

As in other experiments of this nature, a key challenge to causally identifying treatment effects is the difference between treatment assignment and actual treatment exposure. The number of individuals who participated in the chatroom at any length and completed at least part of the post-survey was 1,574. However, the number of those who received the full treatment, meaning they were in chats that went at least 4 AI rephrasings long as intended in the design of the experiment, was 698 (233 Control, 465 treatment). In the main text, we follow Gerber and Green in calculating and presenting results based on placebo-controlled estimations of the treatment effect [3], in which a subset of individuals in the treatment group are compared to individuals in the control group who had a similar length conversation. For reasons identified by Gerber and Green, we believe this to be the best estimation of our treatment effects.

Perhaps the most conservative estimate of the treatment effect is to calculate the effect based solely on *treatment assignment* without accounting for exposure to the treatment in practice. In this case, randomization ensures that differences in the outcome variables between the treatment groups are not linked to any confounders, specifically differential attrition from the treatment and control groups as a result of exposure to the treatment. The 0+ group in Figure 4 in the main text presents this estimate, comparing means for the entire sample regardless of how many rephrasing interventions they received (or, for those in the control condition, how many they would have received had they been in a treated conversation).

However, estimated effect sizes in the 0+ group are problematic because a number of people (approximately 240) who did not, in practice, have any conversation or receive any of the treatment, are included as though they were fully treated. Additionally, the 0+ estimate fails to account for how various treatment dosage levels impact the outcome. Therefore, we also estimate treatment effects conditional on the level of treatment actually received, or "complier average causal effects" CACE using a placebo-controlled approach.

In the placebo-controlled estimates of CACE, we calculate the treatment effect for subgroups of the sample (both treatment and control) who had a conversation long enough to receive one or more rephrasing (1+), two or more rephrasings (2+), three or more rephrasings (3+), and four or more rephrasings (4+). Note that these subgroups are nested, such that all the participants in the 4+ group are included in each lower level as well. Because the placebo groups had conversations of roughly equivalent length, and also faced early exit at comparable rates to the treatment group, these results allow us to make comparisons between treated and control individuals who had conversations of similar length, which separates the effect of having a longer conversation from the effect of receiving more of the treatment. These estimates have an advantage of making no statistical modeling assumptions and, as noted by Gerber and Green [3], are the most accurate measure of dosage treatment effects based on the assumption that the decision to continue having a conversation is unrelated to the treatment (receipt of GPT-3 prompts

by one partner).

4.3 Tests of the Placebo Control CACE Assumption

One of the central features of the analyses presented in the main text is our presentation of results by treatment dosage/exposure, measured by conversation length. As we note in the paper, causal identification of the treatment effect for subgroups that had longer conversations (meaning more treatment exposure) hinges on the assumption that respondents' chat length is unrelated to the treatment condition.

Here we test that assumption by exploring what characteristics, if any, correlate with levels of treatment dosage/exposure. In this analysis, we use a linear regression model to predict the number of rephrasings offered in a conversation (or the number of rephrasings that would have been offered in a control conversation had it been treated) by treatment group assignment, pre-treatment support for gun regulation, age, party ID, gender, race, income, education, employment, region, neighborhood, and game console ownership (a proxy for technology comfort).

As Table S5 indicates, we do find some small but statistically significant correlations between conversation length and respondents' pre-chat survey attitudes, party ID, race, income, and education. *Notably, however, treatment assignment is not a significant predictor of length of conversation.* We measure length of conversation in two ways; in models 1 and 2 and it is the number of rephrasings shown (or the number that would have been shown in a control conversation had it been treated), and in models 3 and 4 it is the total number of messages sent by either partner in the conversation. Note that the average difference in number of messages sent between treatment and control conversations is less than one.

	Dependent variable:			
	Rephrasing Interventions Shown		Total Messages in Convo	
	(1)	(2)	(3)	(4)
Treat: GPT-3 partner	−0.01 (0.10)	−0.05 (0.11)	0.76 (0.58)	0.59 (0.61)
Treat: GPT-3 intervention	0.01 (0.10)	−0.03 (0.11)	0.68 (0.58)	0.51 (0.61)
Gun Control Supporter		0.18 (0.10)$^+$		1.00 (0.57)$^+$
Age		0.01 (0.004)		0.01 (0.02)
Party ID		0.04 (0.02)$^+$		0.21 (0.12)$^+$
Male		0.02 (0.09)		0.45 (0.54)
Non-binary		0.49 (0.51)		1.29 (2.91)
Black		−0.34 (0.15)*		−1.61 (0.84)$^+$
Other Race		0.16 (0.14)		0.84 (0.80)
Employment: Other		0.04 (0.26)		0.90 (1.52)
Employment: Retired		−0.16 (0.21)		−0.78 (1.20)
Employment: Student		−0.30 (0.33)		−1.42 (1.92)
Unemployed - Looking		−0.27 (0.23)		−2.13 (1.32)
Unemployed - Not Looking		−0.53 (0.23)*		−3.12 (1.33)*
Employed - Full time		−0.20 (0.16)		−1.42 (0.93)
Employed - Part time		−0.22 (0.20)		−1.90 (1.13)$^+$
Income		0.04 (0.02)		0.15 (0.14)
Education		0.05 (0.03)$^+$		0.36 (0.15)*
Northeast		0.002 (0.15)		0.28 (0.85)
South		0.10 (0.11)		0.61 (0.63)
West		0.01 (0.15)		0.09 (0.86)
Suburban		−0.003 (0.11)		−0.05 (0.61)
Urban		−0.07 (0.12)		−0.58 (0.71)
Owns Game Console		−0.08 (0.11)		−0.29 (0.62)
Constant	2.56 (0.07)**	1.97 (0.31)**	15.02 (0.41)**	12.54 (1.81)**
Observations	1,574	1,451	1,574	1,451
R^2	0.0000	0.03	0.001	0.03
Adjusted R^2	−0.001	0.01	0.0000	0.01
Residual Std. Error	1.64 (df=1571)	1.64 (df=1426)	9.44 (df=1571)	9.45 (df=1426)
F Statistic	0.01 (df=2; 1571)	1.87** (df=24; 1426)	1.03 (df=2; 1571)	1.78** (df=24; 1426)
Note:				$^+p<0.1$; $^*p<0.05$; $^{**}p<0.01$

Table S5: OLS Models exploring Balanced Attrition Assumption

5 Analysis of the Distribution of Conversational Topics and Rewrites

Figure 3 in the main text considers the content of the messages sent in the chatrooms. To create this visualization and perform this analysis, we converted all 10,695 messages over 4 words long into a 768-dimensional feature vector using the `sentence-tranformers` library and the `sentence-t5-xxl` model. This model is optimized for capturing semantic similarity between sentences. In line with standard practice, these large-dimensional vectors were then reduced to 50 dimensions with PCA and embedded into the 2D space using the UMAP library (with n_neighbors=5, min_dist=0.001, and a cosine similarity metric). The resulting two-dimensional points were then clustered using `scikit-learn`'s K-Means algorithm, which is an unsupervised clustering algorithm. We used default parameters with K=12 (we selected the number of clusters with a standard inertia analysis) . To summarize the content of each cluster without introducing any human bias, we used GPT-4 to automatically generate a synthetic label for the cluster based on a simple prompt. These labels were included as-generated, without any cherry picking or additional text processing. This creates the cluster names shown in Figure 3. Our own manual checking of this clustering supported the labels created by GPT-4.

Panel A of Figure 3 in the main text shows that the topics participants discussed were on the subject we intended (gun regulation), although the specific aspect of gun regulation varied across the messages.

As discussed in the main text, messages were selected for treatment based on turn count; this means that they were essentially selected randomly. Panel B shows each message that was selected for treatment, and that they are uniformly spread out across the semantic space. Panel C shows the distribution of the corresponding rephrased messages, which are again spread uniformly across the space. This suggests that there are no obvious degeneracies in our rewriting algorithm (such as mapping all rewritten messages to the same cluster, or otherwise significantly skewing the topical distribution).

To quantify this, we used the K-Means model to assign each message to a topic cluster, and then calculated topic proportions for the untreated, treated-before-rewrite, and treated-after-rewrite messages; these proportions are shown in Panel D. A simple chi-square test suggests that these distributions are not significantly different (chi-squared, N=871, p=1.00).

Because the rewritten messages do not appear to be statistically different than their original counterparts (from a topic standpoint), we conclude that our rewriting algorithm only affected their tone – the politeness, validation, and restating we intended.

6 Factor Analysis of Index Items

The Methods section describes how we combined various items into index dependent variables. In addition to calculating Cronbach's α, we also conducted exploratory factor analyses for each of these measures. The EFA results lend additional support for the combination of the individual items into the indices used in the paper. Plots for the very simple structure fit of these sets of questions support a single index for each concept, as shown in figures S5, S6, S7, S8, and S9. In these figures, a single factor performs generally as well as a two or more factor solution, confirming the results of the Cronbach's α estimates shown in the Methods section.

Additional details about the specific factor loadings from these analyses can be found in Table S6 for the conversation quality items, Table S7 for the democratic reciprocity items, Table S8 for the gun policy items on the pre-chat survey, Table S9 for the gun policy items on the post-chat survey, and Table S10 for the gun policy items on the follow-up survey three months later.

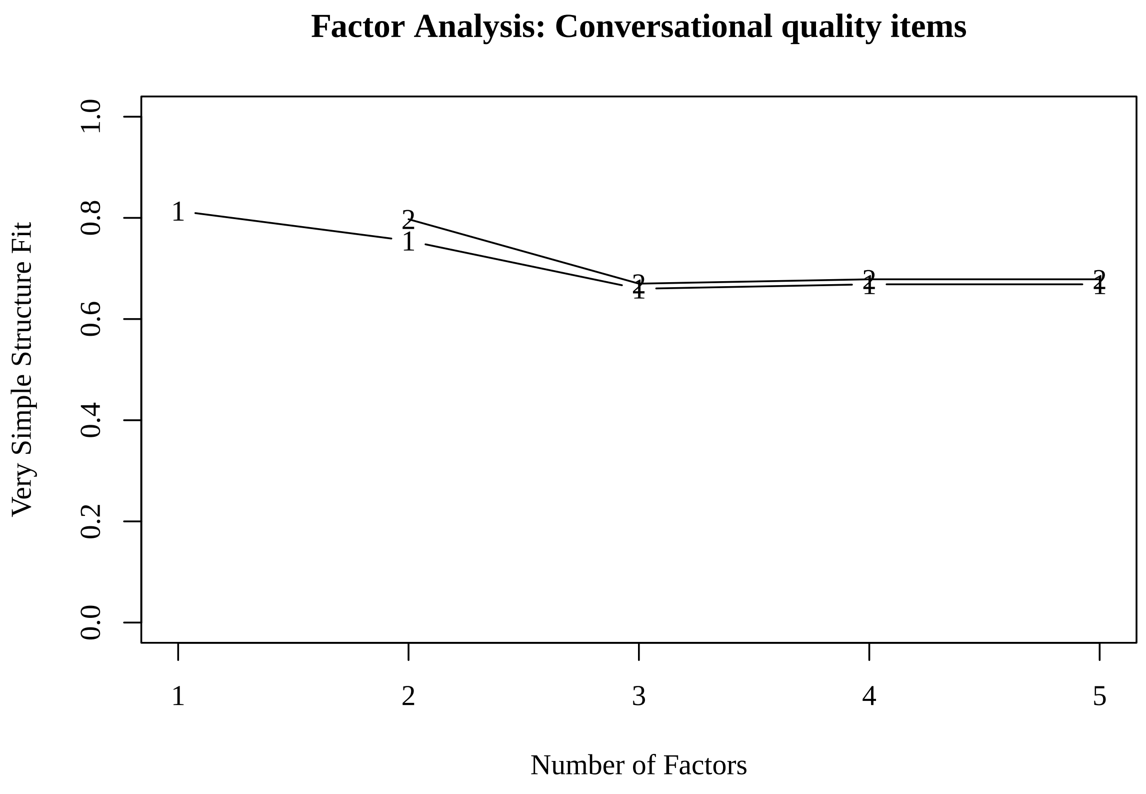

Figure S5: Based on factor analysis of conversation quality items

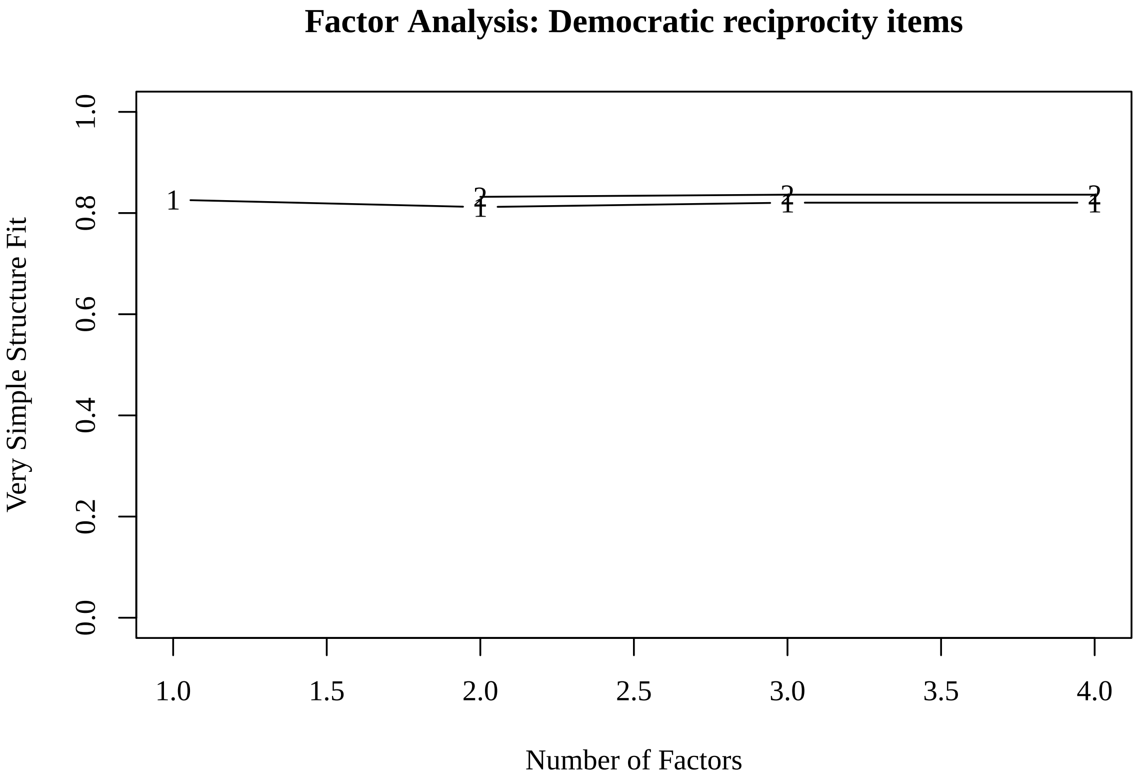

Figure S6: Based on factor analysis of democratic reciprocity items

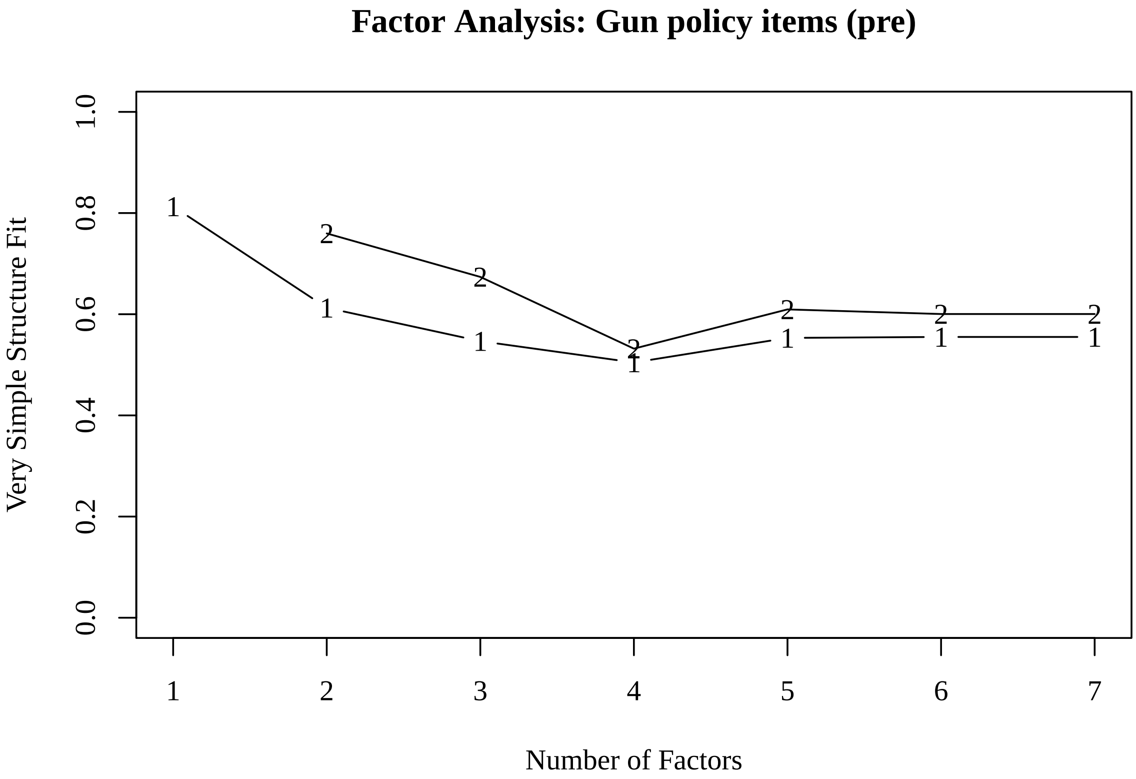

Figure S7: Based on factor analysis of pre-treatment gun policy questions

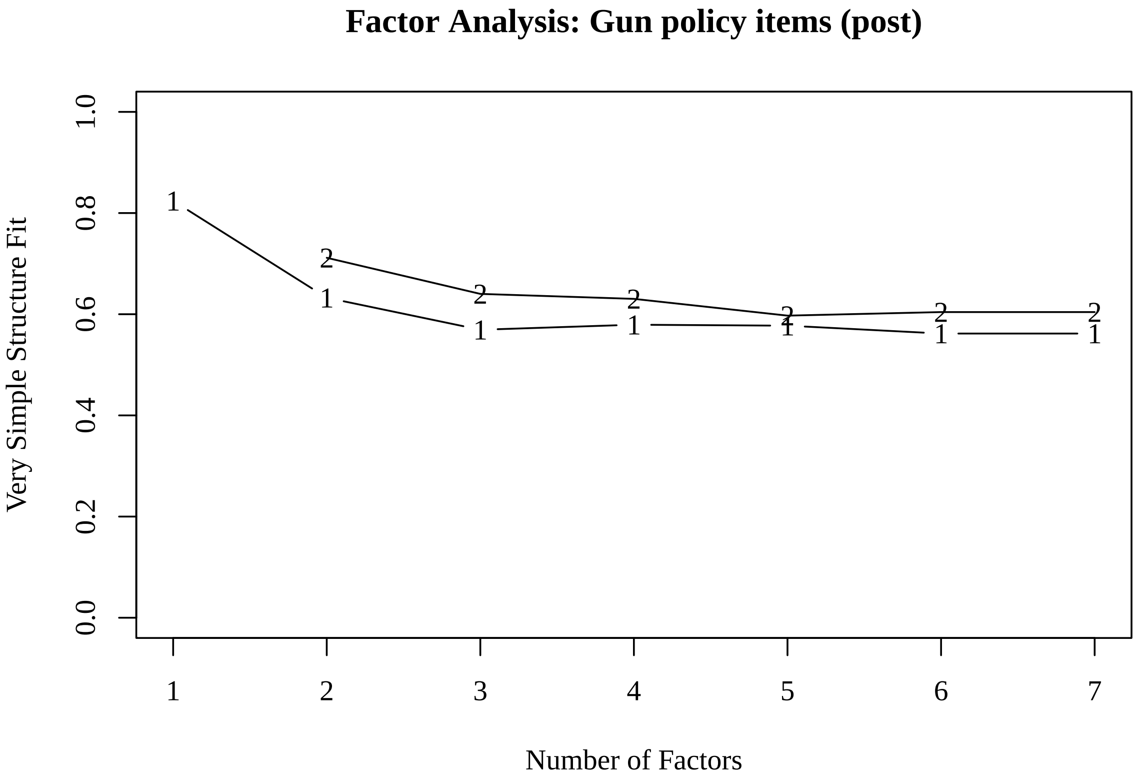

Figure S8: Based on factor analysis of post-treatment gun policy questions

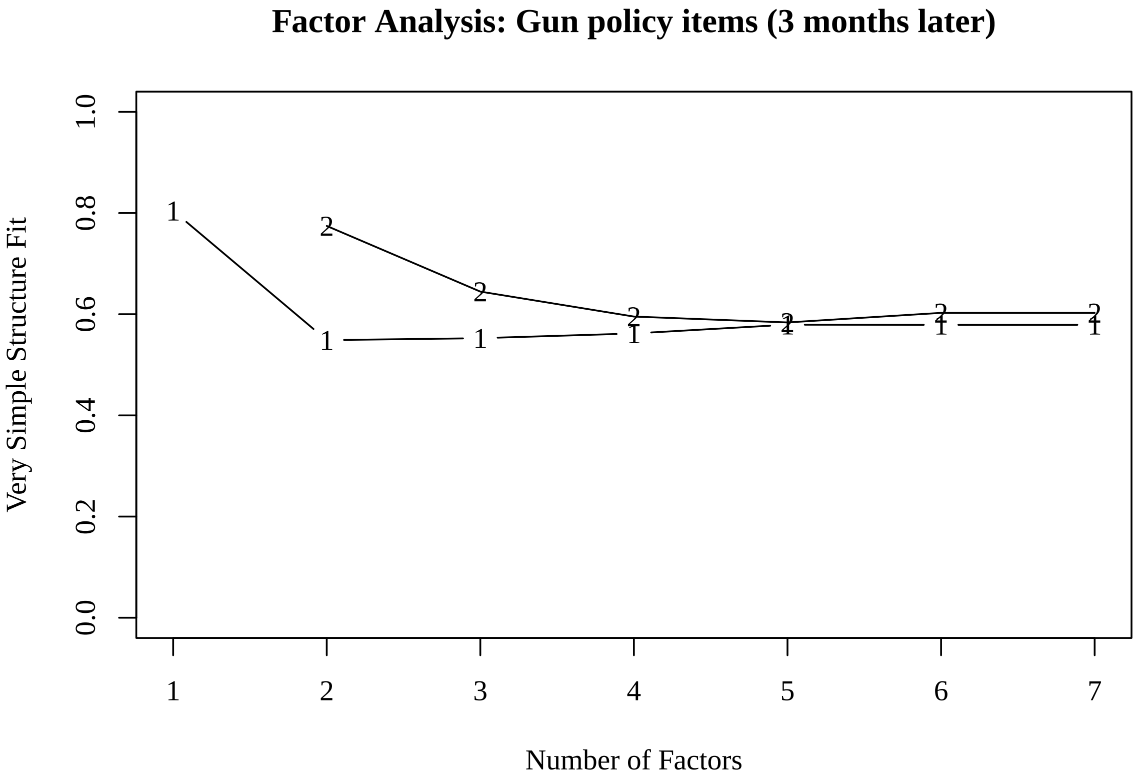

Figure S9: Based on factor analysis of follow-up survey gun policy questions

Item	Loadings
Grade	0.7384
Felt Understood	0.8567
Treated w/ Respect	0.4724
Disrespectful	0.5279
Communicate	0.7176

Table S6: Factor loadings for a one-factor exploratory factor analysis of the conversation items. All items are scored so that higher values indicate better conversations. Cronbach's α for this index was 0.79.

Item	Loadings
Point of view	0.4757
Perspective	0.7398
Good reasons	0.7774
Respect	0.782

Table S7: Factor loadings for a one-factor exploratory factor analysis of the democratic reciprocity items. All items are scored so that higher values indicate more democratic reciprocity. Cronbach's α was 0.78.

Item	Loadings
Mental Illness Purchases	0.4881
Ban Assault-style	0.8424
Ban High-capacity	0.8314
Carry Concealed	0.5644
Arm Teachers	0.4474
Enhanced Background checks	0.6142
Red Flag Laws	0.7256

Table S8: Factor loadings for a one-factor exploratory factor analysis of the gun policy items on the pre-chat survey. All items are scored so that higher values indicate more support for gun restrictions. Cronbach's α was 0.83.

Item	Loadings
Mental Illness Purchases	0.5397
Ban Assault-style	0.8404
Ban High-capacity	0.8414
Carry Concealed	0.5741
Arm Teachers	0.4025
Enhanced Background checks	0.6535
Red Flag Laws	0.7244

Table S9: Factor loadings for a one-factor exploratory factor analysis of the gun policy items on the post-chat survey. All items are scored so that higher values indicate more support for gun restrictions. Cronbach's α was 0.83.

Item	Loadings
Mental Illness Purchases	0.4693
Ban Assault-style	0.8626
Ban High-capacity	0.8258
Carry Concealed	0.5661
Arm Teachers	0.4182
Enhanced Background checks	0.6008
Red Flag Laws	0.7198

Table S10: Factor loadings for a one-factor exploratory factor analysis of the gun policy items on the follow-up survey about three months later. All items are scored so that higher values indicate more support for gun restrictions. Cronbach's α was 0.82.

7 Treatment Effects by Item

In the main text, we rely on a series of indices for our analyses instead of examining the constituent items individually. We do this for the conversation quality, democratic reciprocity, and attitudes about gun policy. This approach recognizes the various benefits provided by relying on multi-item scales [1, 2], and is supported by the psychometric properties of these items as described in Section 6. We also rely on these indices for ease and simplicity of presentation in the main text of the paper.

In this part of the appendix, we present the results separately for each item that makes up each index. For each index, we first present a figure that graphs the means and 95% confidence intervals for each variable by treatment condition for each subgroup in our analysis. We then present a numerical table of the means and standard deviations used to create those figures. For just the Democratic Reciprocity items, we also present linear regression models which provide an alternate statistical test of the difference in means (placebo-control CACE) displayed in the figures.

7.1 Conversation Quality Index

		Control		GPT-3 Self				GPT-3 Partner			
		Mean	n	Mean	n	Diff	p	Mean	n	Diff	p
1	0+	0.76	530	0.76	521	0.00	0.80	0.79	523	0.03	0.03
2	1+	0.78	478	0.79	448	0.01	0.48	0.82	447	0.04	0.00
3	2+	0.82	330	0.83	343	0.00	0.75	0.85	345	0.03	0.05
4	3+	0.83	283	0.84	280	0.01	0.60	0.86	277	0.03	0.02
5	4+	0.83	233	0.84	233	0.01	0.57	0.87	232	0.04	0.01

Table S11: Conversation Index T-Tests: Columns indicate the mean and sample size for the conversation quality index by treatment condition. The mean difference and p-value for the treatment conditions are relative to the control condition, and are derived from a standard independent-samples t-test. These are the significance levels reported in the main text Figure 4.

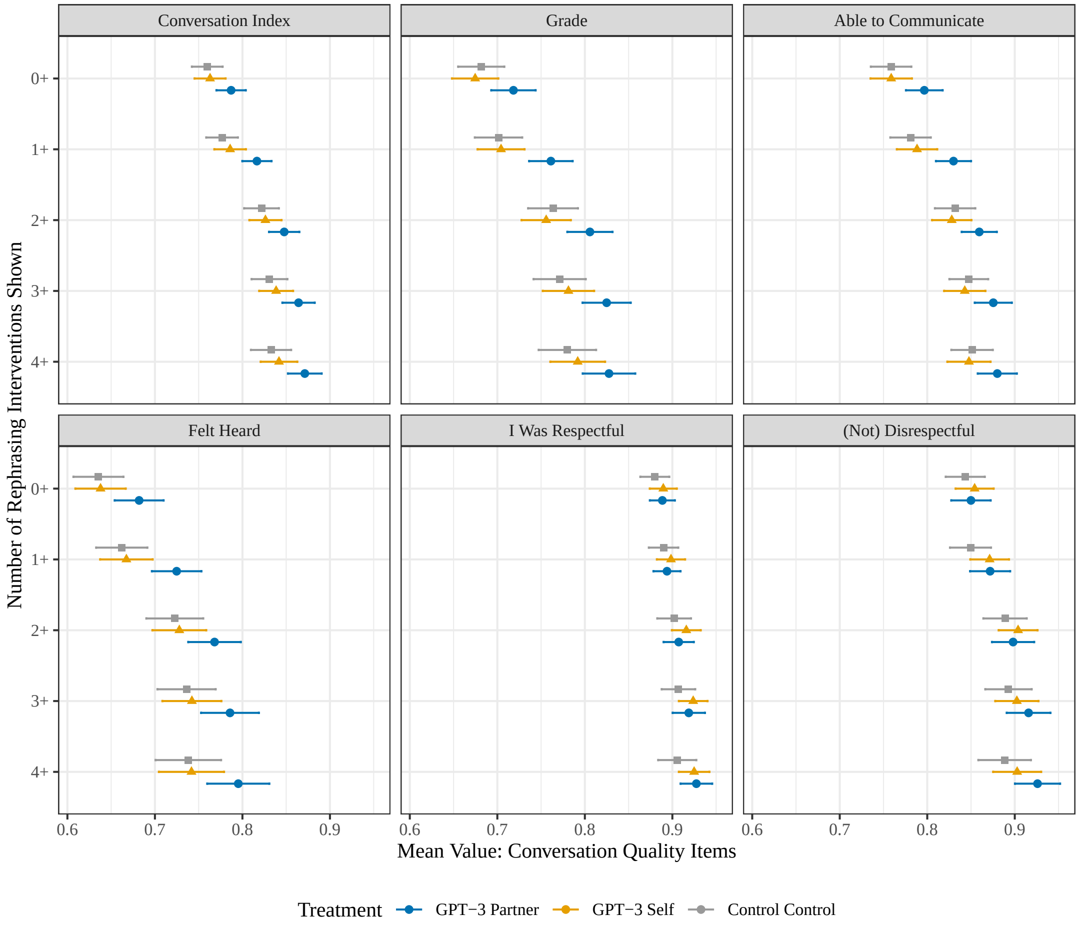

Figure S10: Placebo-control CACE Effects: Mean and 95% confidence interval for all metrics that make up the conversation quality index, by treatment condition and conversation length.

	DV	Treatment	0+ mean	0+ sd	1+ mean	1+ sd	2+ mean	2+ sd	3+ mean	3+ sd	4+ mean	4+ sd
1	Conversation Index	Control	0.76	0.21	0.78	0.20	0.82	0.18	0.83	0.18	0.83	0.18
2	Conversation Index	Partner	0.79	0.20	0.82	0.18	0.85	0.16	0.86	0.16	0.87	0.15
3	Conversation Index	Self	0.76	0.21	0.79	0.19	0.83	0.17	0.84	0.17	0.84	0.16
4	Grade	Control	0.68	0.31	0.70	0.30	0.76	0.26	0.77	0.26	0.78	0.25
5	Grade	Partner	0.72	0.30	0.76	0.27	0.81	0.24	0.82	0.23	0.83	0.23
6	Grade	Self	0.67	0.31	0.70	0.29	0.76	0.27	0.78	0.25	0.79	0.24
7	Able to Communicate	Control	0.76	0.27	0.78	0.26	0.83	0.21	0.85	0.19	0.85	0.18
8	Able to Communicate	Partner	0.80	0.24	0.83	0.22	0.86	0.19	0.88	0.18	0.88	0.17
9	Able to Communicate	Self	0.76	0.28	0.79	0.25	0.83	0.21	0.84	0.20	0.85	0.19
10	Felt Heard	Control	0.64	0.34	0.66	0.33	0.72	0.30	0.74	0.28	0.74	0.29
11	Felt Heard	Partner	0.68	0.33	0.72	0.30	0.77	0.28	0.79	0.28	0.80	0.27
12	Felt Heard	Self	0.64	0.33	0.67	0.32	0.73	0.29	0.74	0.29	0.74	0.29
13	I Was Respectful	Control	0.88	0.19	0.89	0.19	0.90	0.18	0.91	0.16	0.91	0.17
14	I Was Respectful	Partner	0.89	0.17	0.89	0.17	0.91	0.16	0.92	0.16	0.93	0.14
15	I Was Respectful	Self	0.89	0.18	0.90	0.17	0.92	0.16	0.92	0.14	0.92	0.14
16	(Not) Disrespectful	Control	0.84	0.26	0.85	0.26	0.89	0.23	0.89	0.23	0.89	0.23
17	(Not) Disrespectful	Partner	0.85	0.26	0.87	0.25	0.90	0.23	0.92	0.21	0.93	0.20
18	(Not) Disrespectful	Self	0.85	0.25	0.87	0.24	0.90	0.21	0.90	0.21	0.90	0.21

Table S12: Conversation Index Item Means: Columns indicate the mean and standard deviation for the conversation quality index, followed by each constituent item, by subsets of the minimum number of rephrasings that was shown to the treated respondent (or that would have been shown in a control condition if it had been treated.)

7.2 Democratic Reciprocity Index

		Control		GPT-3 Self				GPT-3 Partner			
		Mean	n	Mean	n	Diff	p	Mean	n	Diff	p
1	0+	0.70	530	0.70	521	0.01	0.56	0.71	523	0.02	0.19
2	1+	0.70	478	0.71	448	0.01	0.39	0.73	447	0.03	0.05
3	2+	0.70	330	0.73	343	0.03	0.04	0.74	345	0.04	0.02
4	3+	0.70	283	0.74	280	0.04	0.01	0.75	277	0.05	0.00
5	4+	0.70	233	0.74	233	0.04	0.02	0.76	232	0.06	0.00

Table S13: Democratic Reciprocity Index T-Tests: Columns indicate the mean and sample size for the reciprocity index by treatment condition. The mean difference and p-value for the treatment conditions are relative to the control condition, and are derived from a standard independent-samples t-test. These are the significance levels reported in the main text Figure 4.

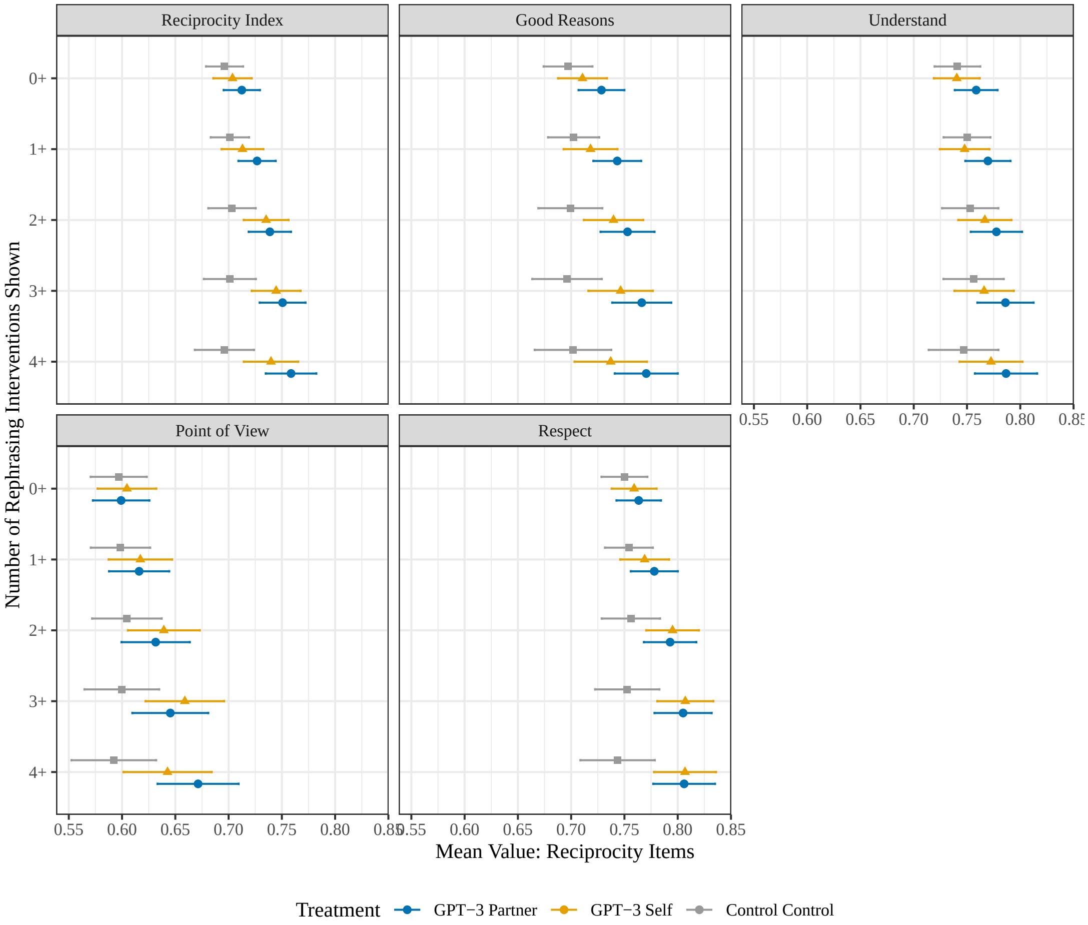

Figure S11: Mean and 95% confidence interval for all metrics that make up the democratic reciprocity index, by treatment condition and conversation length.

	DV	Treatment	0+ mean	0+ sd	1+ mean	1+ sd	2+ mean	2+ sd	3+ mean	3+ sd	4+ mean	4+ sd
1	Reciprocity Index	Control	0.70	0.20	0.70	0.20	0.70	0.21	0.70	0.21	0.70	0.22
2	Reciprocity Index	Partner	0.71	0.20	0.73	0.19	0.74	0.19	0.75	0.18	0.76	0.18
3	Reciprocity Index	Self	0.70	0.21	0.71	0.21	0.74	0.20	0.74	0.20	0.74	0.20
4	Good Reasons	Control	0.70	0.27	0.70	0.27	0.70	0.28	0.70	0.28	0.70	0.28
5	Good Reasons	Partner	0.73	0.25	0.74	0.24	0.75	0.24	0.77	0.23	0.77	0.23
6	Good Reasons	Self	0.71	0.27	0.72	0.27	0.74	0.26	0.75	0.26	0.74	0.26
7	Point of View	Control	0.60	0.31	0.60	0.31	0.60	0.30	0.60	0.30	0.59	0.31
8	Point of View	Partner	0.60	0.31	0.62	0.30	0.63	0.30	0.65	0.30	0.67	0.29
9	Point of View	Self	0.60	0.32	0.62	0.32	0.64	0.32	0.66	0.31	0.64	0.32
10	Understand	Control	0.74	0.25	0.75	0.24	0.75	0.24	0.76	0.24	0.75	0.25
11	Understand	Partner	0.76	0.23	0.77	0.23	0.78	0.23	0.79	0.22	0.79	0.23
12	Understand	Self	0.74	0.25	0.75	0.25	0.77	0.24	0.77	0.24	0.77	0.23
13	Respect	Control	0.75	0.25	0.75	0.25	0.76	0.25	0.75	0.26	0.74	0.27
14	Respect	Partner	0.76	0.24	0.78	0.24	0.79	0.23	0.81	0.23	0.81	0.22
15	Respect	Self	0.76	0.25	0.77	0.25	0.80	0.23	0.81	0.22	0.81	0.23

Table S14: Reciprocity Index Means: Columns indicate the mean and standard deviation for the dreciprocity index, followed by each constituent item, by subsets of the minimum number of rephrasings that was shown to the treated respondent (or that would have been shown in a control condition if it had been treated.)

For the Democratic Reciprocity Index and each of the constituent variables, we also provide a simple linear regression model that predicts each outcome with the treatment assignment as the sole independent variable. This functions as a statistical test of the mean differences visualized in Figure 4 in the main paper.

	Dependent variable:				
	Reciprocity Index				
	0+	1+	2+	3+	4+
Partner	0.02	0.03^{+}	0.04^{*}	0.05^{**}	0.06^{**}
	(0.01)	(0.01)	(0.02)	(0.02)	(0.02)
Self	0.01	0.01	0.03^{*}	0.04^{**}	0.04^{*}
	(0.01)	(0.01)	(0.02)	(0.02)	(0.02)
Constant	0.70^{**}	0.70^{**}	0.70^{**}	0.70^{**}	0.70^{**}
	(0.01)	(0.01)	(0.01)	(0.01)	(0.01)
Observations	1,572	1,371	1,017	840	698
R^2	0.001	0.003	0.01	0.01	0.02
Adjusted R^2	−0.0002	0.001	0.004	0.01	0.01
Note:	$^{+}$p<0.1; *p<0.05; **p<0.01				

Table S15: Reciprocity Index OLS Models

	Dependent variable:				
	Good Reasons				
	0+	1+	2+	3+	4+
Partner	0.03^{+}	0.04^{*}	0.05^{**}	0.07^{**}	0.07^{**}
	(0.02)	(0.02)	(0.02)	(0.02)	(0.02)
Self	0.01	0.02	0.04^{*}	0.05^{*}	0.04
	(0.02)	(0.02)	(0.02)	(0.02)	(0.02)
Constant	0.70^{**}	0.70^{**}	0.70^{**}	0.70^{**}	0.70^{**}
	(0.01)	(0.01)	(0.01)	(0.02)	(0.02)
Observations	1,572	1,371	1,017	840	698
R^2	0.002	0.004	0.01	0.01	0.01
Adjusted R^2	0.001	0.003	0.01	0.01	0.01
Note:	$^{+}$p<0.1; *p<0.05; **p<0.01				

Table S16: Good Reasons OLS Models

	Dependent variable:				
	Understand				
	0+	1+	2+	3+	4+
Partner	0.02	0.02	0.02	0.03	0.04^{+}
	(0.02)	(0.02)	(0.02)	(0.02)	(0.02)
Self	−0.001	−0.002	0.01	0.01	0.03
	(0.02)	(0.02)	(0.02)	(0.02)	(0.02)
Constant	0.74^{**}	0.75^{**}	0.75^{**}	0.76^{**}	0.75^{**}
	(0.01)	(0.01)	(0.01)	(0.01)	(0.02)
Observations	1,572	1,371	1,017	840	698
R^2	0.001	0.002	0.002	0.003	0.005
Adjusted R^2	−0.0001	0.0002	−0.0002	0.0004	0.002
Note:	$^{+}$p<0.1; *p<0.05; **p<0.01				

Table S17: Understand OLS Models

	Dependent variable:				
	Point of View				
	0+	1+	2+	3+	4+
Partner	0.002	0.02	0.03	0.05^{+}	0.08^{**}
	(0.02)	(0.02)	(0.02)	(0.03)	(0.03)
Self	0.01	0.02	0.03	0.06^{*}	0.05^{+}
	(0.02)	(0.02)	(0.02)	(0.03)	(0.03)
Constant	0.60^{**}	0.60^{**}	0.60^{**}	0.60^{**}	0.59^{**}
	(0.01)	(0.01)	(0.02)	(0.02)	(0.02)
Observations	1,572	1,371	1,017	840	698
R^2	0.0001	0.001	0.002	0.01	0.01
Adjusted R^2	−0.001	−0.001	0.0003	0.005	0.01
Note:	$^{+}$p<0.1; *p<0.05; **p<0.01				

Table S18: Point of View OLS Models

	Dependent variable:				
	Respect				
	0+	1+	2+	3+	4+
Partner	0.01	0.02	0.04^{*}	0.05^{**}	0.06^{**}
	(0.02)	(0.02)	(0.02)	(0.02)	(0.02)
Self	0.01	0.01	0.04^{*}	0.05^{**}	0.06^{**}
	(0.02)	(0.02)	(0.02)	(0.02)	(0.02)
Constant	0.75^{**}	0.75^{**}	0.76^{**}	0.75^{**}	0.74^{**}
	(0.01)	(0.01)	(0.01)	(0.01)	(0.02)
Observations	1,572	1,371	1,017	840	698
R^2	0.001	0.002	0.01	0.01	0.01
Adjusted R^2	−0.001	0.0002	0.004	0.01	0.01
Note:	$^{+}$p<0.1; *p<0.05; **p<0.01				

Table S19: Respect OLS Models

7.3 Change in Gun Policy Attitudes

Figure S12 presents estimated treatment effects on the absolute value of the change in an index of gun policy positions from before the conversation to after the conversation. Because we do not expect the conversations, which are balanced between matched pairs of gun control supporters and opponents, to result in general movement to the right or the left, we examine the absolute value of change in responses. Lower values indicate less movement in attitudes. As Figure S12 illustrates, we find no evidence of treatment effects on individuals' post-chat gun control policy positions. GPT-3 rephrasings do not seem to increase persuadability on the issues, even while they make the people and arguments on the other side appear more reasonable. This provides evidence that these kinds of AI tools can be used to improve divisive political conversations without manipulating respondents to adopt a particular political viewpoint.

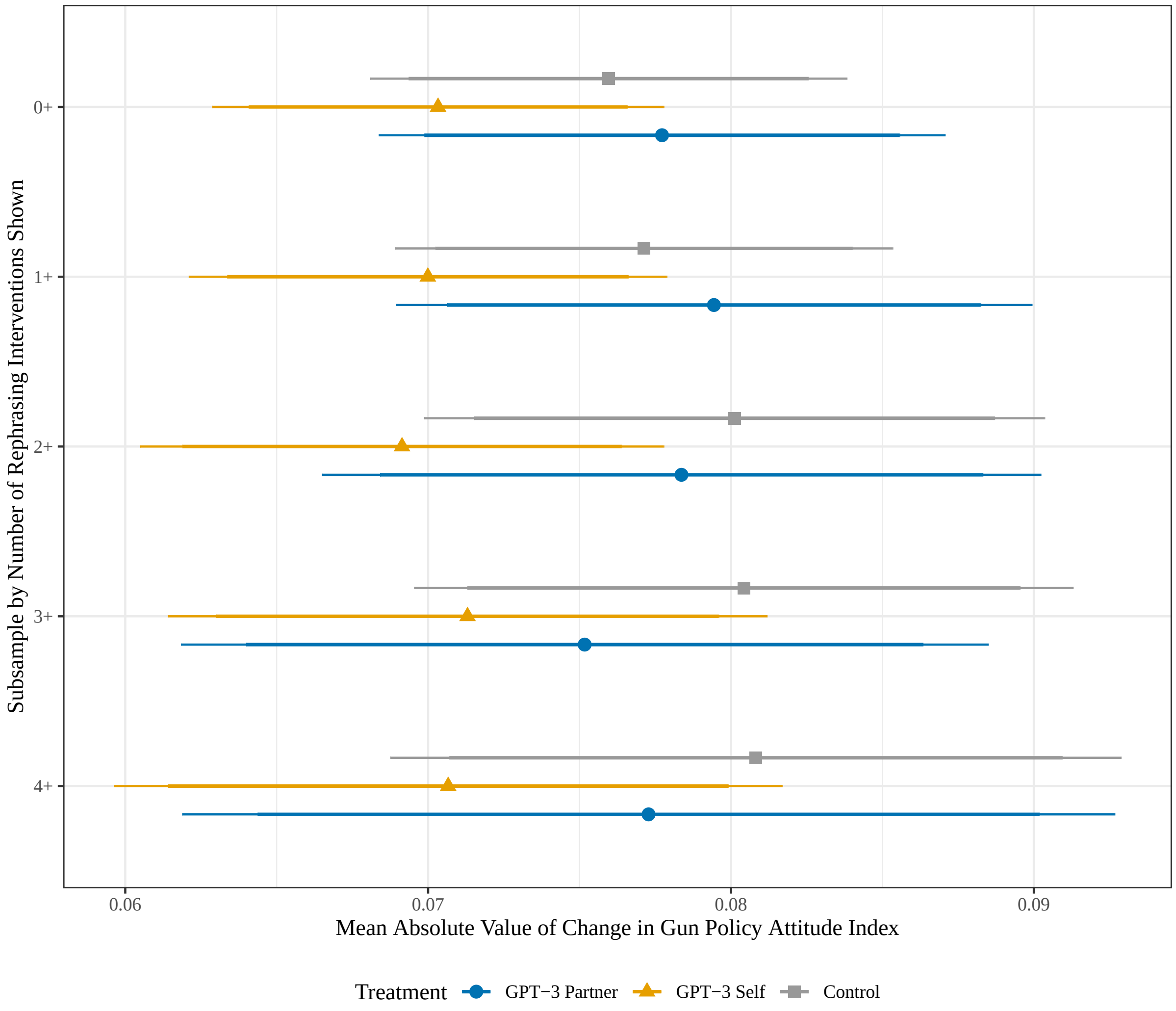

Figure S12: Analysis of Change in Gun Policy Attitudes. The index is scaled from 0 (no change in attitude) to 1 (complete reversal of opinions), and is the absolute value of the post-survey scale value minus the pre-survey scale value. The number of rephrasings are overlapping sets, such that 0+ includes all observations. The means and 95% confidence intervals are based on unadjusted standard errors.

7.4 Over Time Results

As noted in the main text, we sent a follow-up survey to the original study participants approximately 3 months after the chat experiment. We asked respondents to answer the same questions to measure democratic reciprocity. To evaluate the persistence of these effects, we re-ran the main treatment analyses with these follow-up measures. We estimated these only for the democratic reciprocity index as we did not observe effects on the gun policy items during the original experiment and we did not ask the conversation quality questions on the follow-up (as it made little sense with the passage of time to do so). Figure S13 displays these results, showing no evidence of persistence of the key effects noted in our study. Given the fleeting, one-time nature of our treatment, this is not surprising.

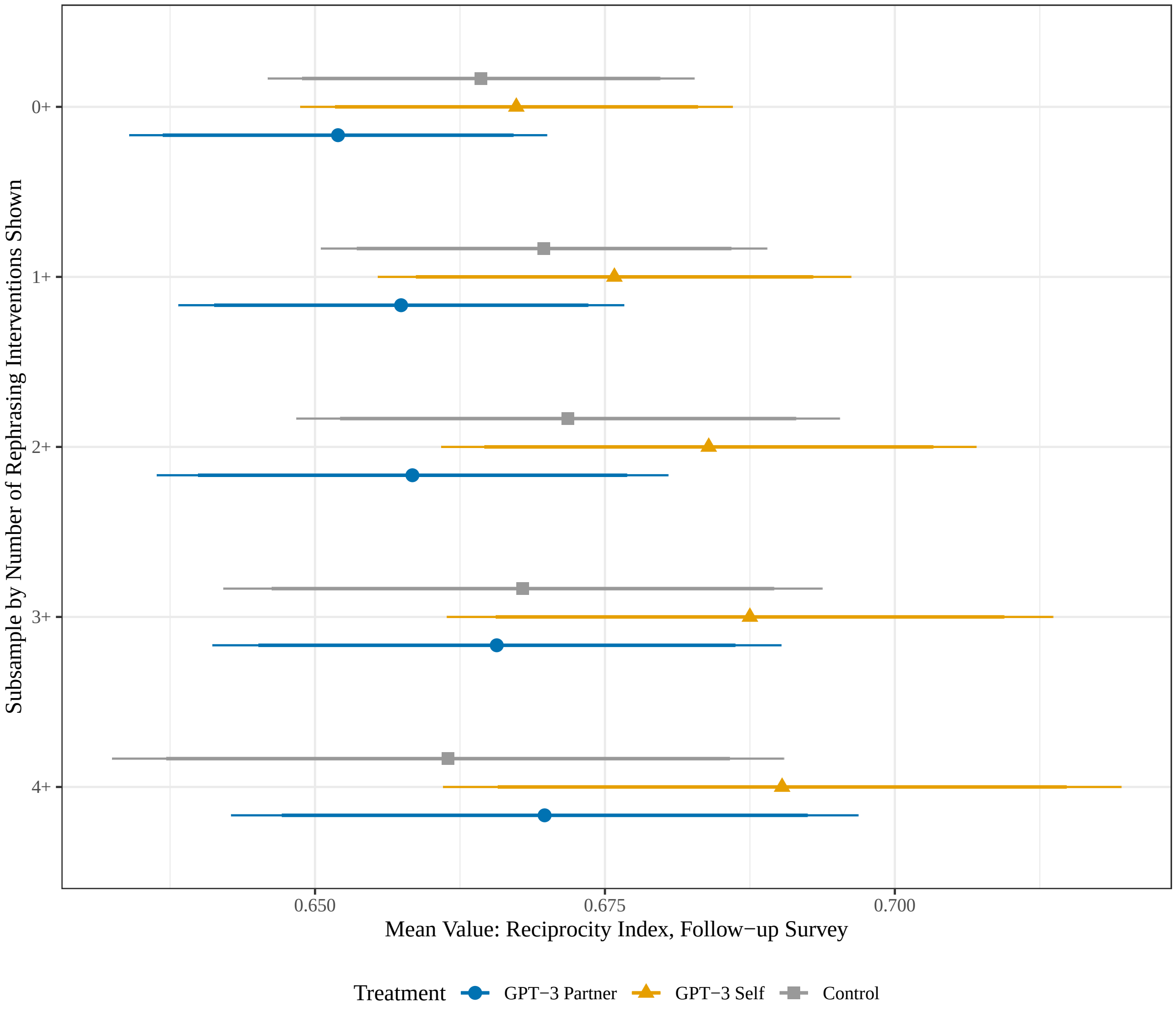

Figure S13: Analysis of democratic reciprocity index on the follow-up survey. The index is scaled from 0 (lowest level of democratic reciprocity) to 1 (highest democratic reciprocity). The number of rephrasings are overlapping sets, such that 0+ includes all observations. The left panel presents the means, 90% and 95% confidence intervals based on unadjusted standard errors. We observe no treatment effects approximately 3 months after the original experiment.

8 Heterogeneous Treatment Effects by Level of Partner Disagreement

One additional question relevant to the generalizability of these results is whether the treatment is more effective in contexts where there is a high level or a low level of pre-existing disagreement between discussion partners. We evaluate these heterogeneous treatment effects in this section. A few caveats: these analyses are not pre-registered, the sample sizes are too small to robustly estimate the statistical significance of effects on smaller subsets of the data, and the level of disagreement is a non-randomized pre-treatment covariate. Therefore, levels of statistical significance and causal inference should be interpreted cautiously. However, we believe that these analyses present an informative impression that the treatment is most effective in situations where there is a higher level of pre-existing disagreement, which provides further confidence that it could be a useful intervention for improving discussions where there exists a high potential for conflict.

8.1 Descriptive Statistics About Partner Disagreement

There are two ways in which we identify people's attitudes about gun control, a single sorting question and a 7-item index adapted from pew-research (see Table S8 for factor loadings).

For ease in the randomization process, we use a single pre-registered question to sort people into two camps: those who support additional gun regulation, which is the modal position in American politics at the time of the study, and those who oppose additional gun regulation, which includes both people who think the United States has the right amount already and people who think the United States should relax existing gun restrictions. The position preferring LESS gun regulation is a minority view in the public and in our study sample. In the end after the matching process, 606 people were in a conversation with the maximum difference (Supporters of more regulation paired with supporters of less regulation), and 1478 people had a conversation with a more moderate difference (supporters of more regulation paired with people who think current levels are about right.) Due to a technical error, 5 conversations (10 participants) were in conversations in which they had the same view as their partner. These conversations are still included in the main analysis. Only 147 people changed their answer on this question between the pre-survey and the post-survey.

The second metric for evaluating opinions about gun policy is the 7-item index, which provides a more detailed and sensitive measure of policy attitudes. We scale these items together so that they range from 0 to 1, where 1 indicates the strongest support of increased gun control across all items. A second way of measuring the level of disagreement between partners is to take the absolute value of the difference between the pre-survey policy index scores for each partner. The mean difference between partners on the pre-survey is 0.36, with a standard deviation of .22.

There are substantial differences in the average value of the index for subgroups created by the single-item sorting variable. Table S20 provides the mean value of the index variable for each subgroup. The sorting variable appears to reflect meaningful differences in attitudes, as measured by the index. Additionally, the differences between groups are sizeable enough that we can expect meaningful disagreement between both configurations of paired respondents.

Summary Attitude	Mean	N	SD
More	0.81	1060	0.17
Same	0.53	746	0.17
Less	0.34	304	0.20

Table S20: Gun Policy Index Mean by Gun Policy Sorting Question Values

8.2 Randomization Balance by Disagreement

One concern is that the treatment effect in the main text could be dependent on the level of disagreement present between partners prior to the start of the conversation. This is a threat to causal inference if disagreement is imbalanced between the treatment and control conditions. We first provide brief evidence that randomization is appropriately balanced, and the average disagreement present between pairs is comparable, both across treatment conditions and across lengths of the conversation. Furthermore, it could affect the interpretation of the results if longer conversations or conversations with larger treatment effects are those in which there is less disagreement present between the partners to start with.

In the following tables, we present the number of respondents by treatment condition and level of disagreement (using the single sorting variable - Table S21), the mean level of disagreement on gun control by treatment condition (using the gun control index variable - Table S22), and the mean level of disagreement on gun control by conversation length (using the gun control index variable - Table S23). These tables collectively demonstrate that disagreement was evenly balanced across treatment conditions and, if anything, the conversations that persisted are those with more disagreement rather than less.

	GPT-3 intervention	GPT-3 partner	Untreated
Pair: More Reg. with Less Reg.	151	153	154
Pair: More Reg. with Stay the Same	363	367	368

Table S21: Case Counts by Treatment and Level of Disagreement

Treatment	Mean Disagreement	N	SD
GPT-3 intervention	0.36	521	0.22
GPT-3 partner	0.36	523	0.22
Untreated	0.36	530	0.22

Table S22: Average Gun Policy Index Disagreement by Treatment Condition

Rephrasing Interventions	Mean Disagreement	N	SD
0	0.34	201	0.22
1	0.34	355	0.22
2	0.35	178	0.23
3	0.33	142	0.20
4+	0.38	698	0.22

Table S23: Average Gun Policy Index Disagreement by Conversation Length

8.3 Heterogeneous Effects by Disagreement

We now evaluate whether the magnitude of the treatment effect varies based on the level of disagreement present in the pairs prior to the start of the conversation. These results are based on a linear model, similar to those presented in Tables S15 to S19, but now including an interaction between the tripartite treatment condition (Control, Partner, and Self), and the level of disagreement between the pairs. We conduct this analysis using both the binary indicator using the sorting variable (Figures S14 and S16) and the quantitative measure of disagreement based on the absolute value of the difference between the partners on the pre-survey index items (Figures S15 and S17). For ease of interpretation of complex interaction coefficients, we present the predicted values for each model (at first, second, and third quartile values for the index metric) in figures comparable to those in the main text. S14 and S15 show the results for the Conversation Quality Index, and Figures S16 and S17 show the results for the Democratic Reciprocity Index. In all cases, the effect is strongest for participants in conversations where there is the highest level of disagreement. Additionally, for respondents in conversations with lower levels of disagreement, there is a higher baseline rate of reported Conversation Quality and Democratic Reciprocity, but the treatment effect is not statistically significant. This indicates that the aggregate effect documented in the main text is not an artifact of respondents who are already highly agreeable, but rather suggests that the treatment is most effective in the most divisive conversations. However, we note again the caveats that this analysis is not pre-registered nor randomized, and therefore we should interpret such results as suggestive and informative but not definitively causal.

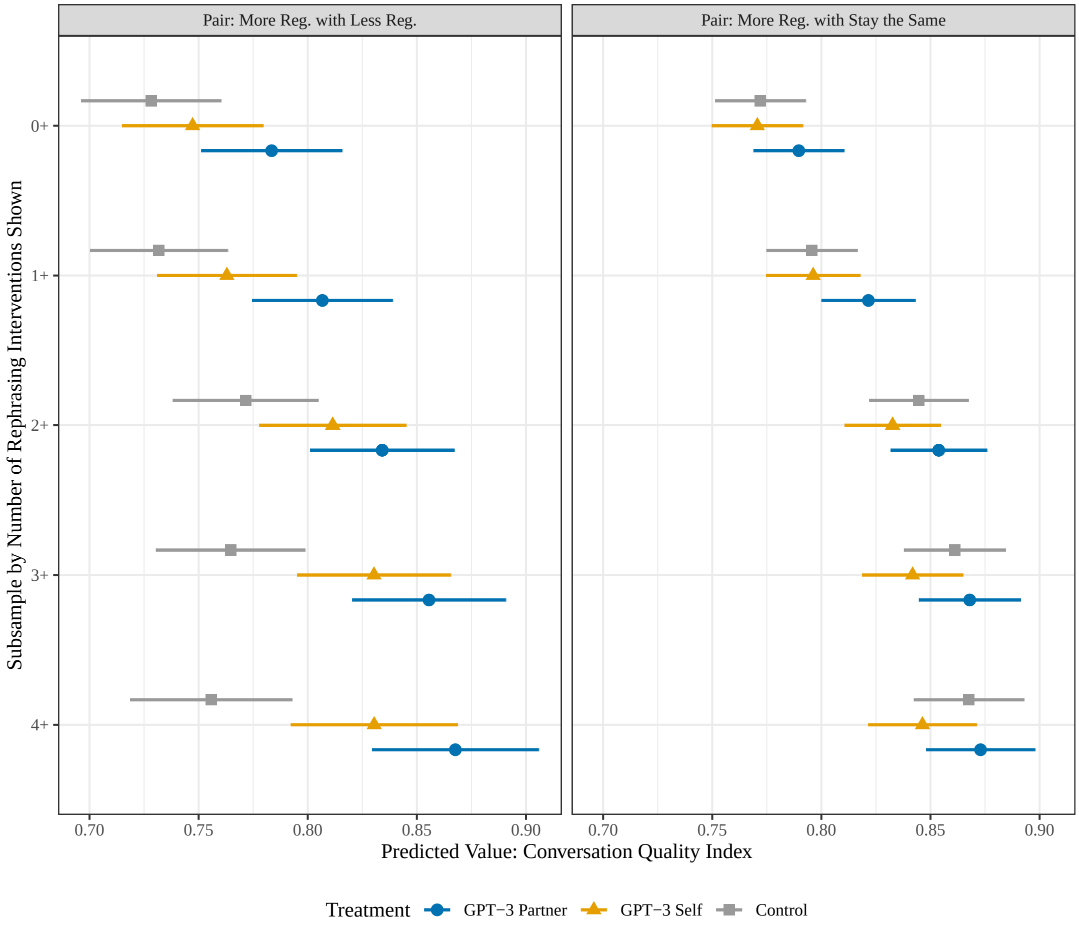

Figure S14: Predicted values of the Conversation Quality Index value, based on an OLS model with an interaction between the treatment condition and the level of partner disagreement (more regulation paired with less regulation, or more regulation paired with stay the same). Models run separately for each conversation length subgroup (0+ to 4+).

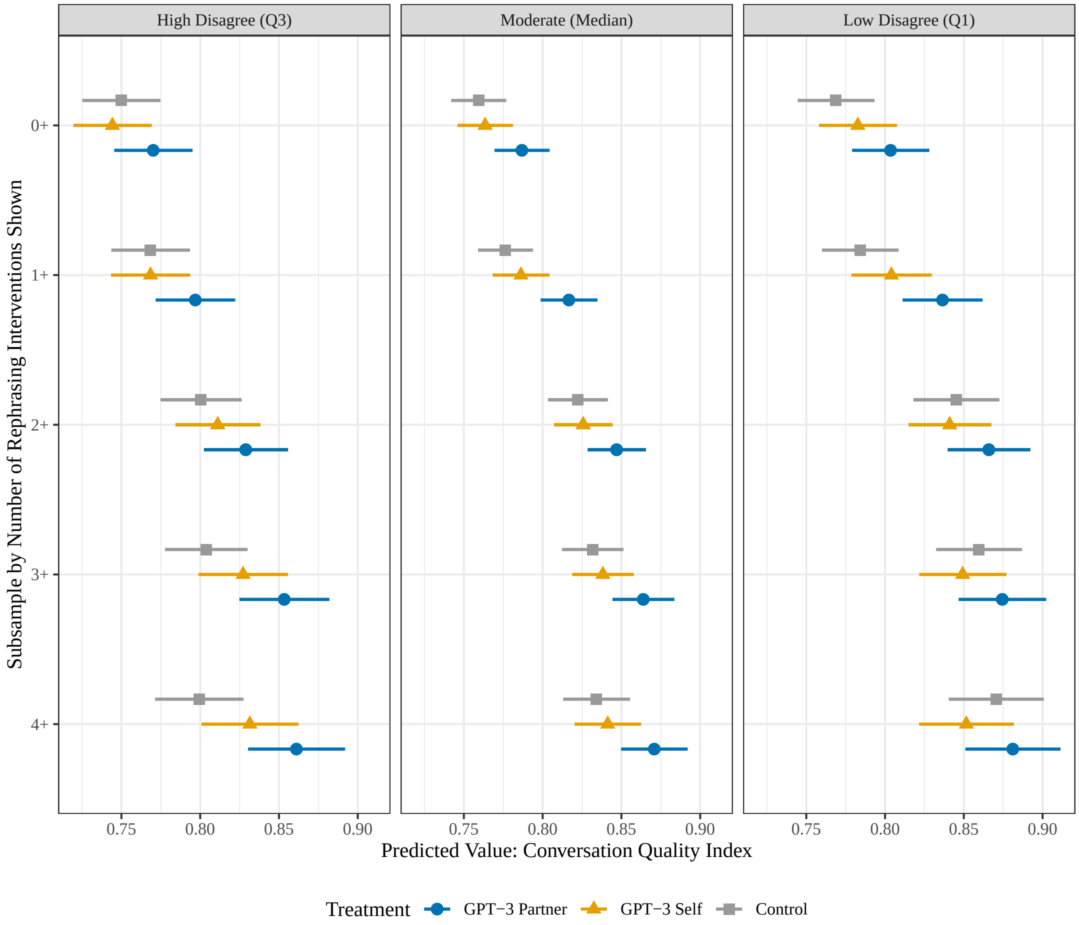

Figure S15: Predicted values of the Conversation Quality Index value, based on an OLS model with an interaction between the treatment condition and the level of partner disagreement. Predicted values shown at quartile values of the difference in pre-survey gun policy attitude indices. Models run separately for each conversation length subgroup (0+ to 4+).

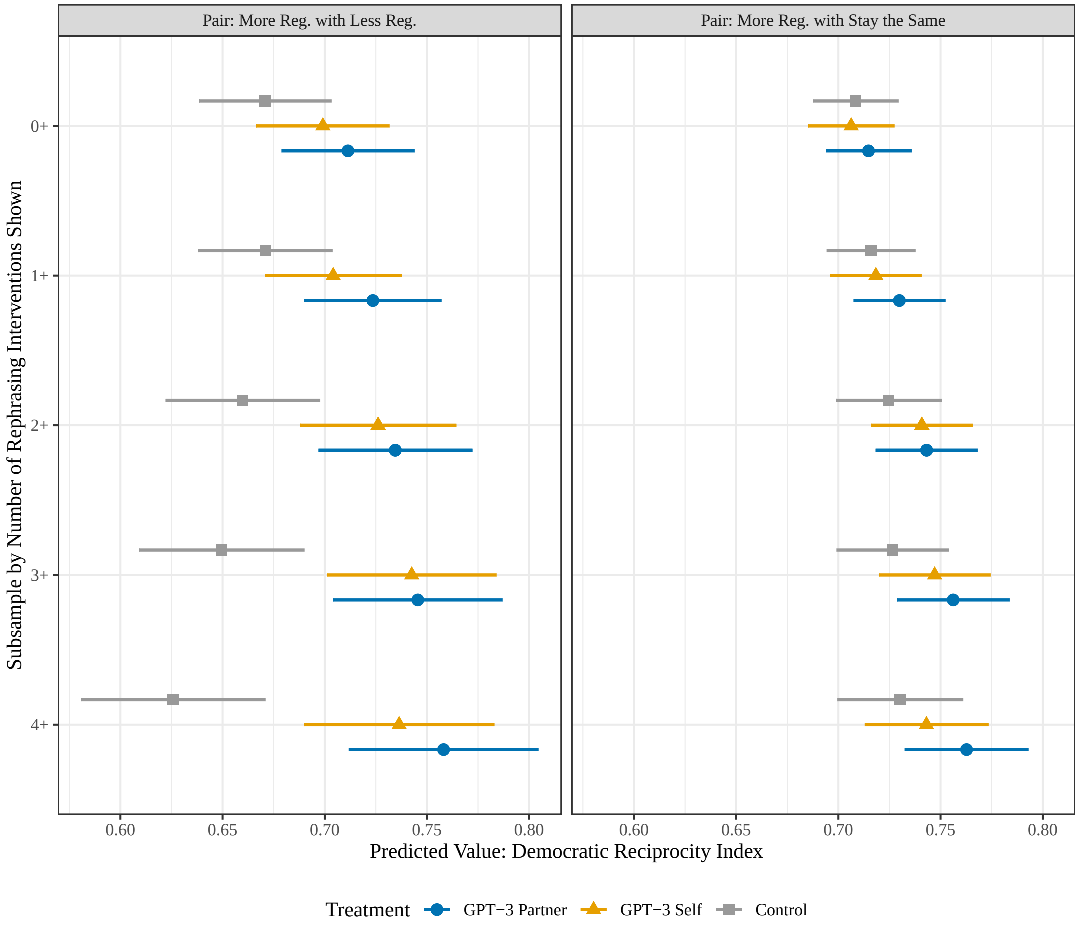

Figure S16: Predicted values of the Democratic Reciprocity Index value, based on an OLS model with an interaction between the treatment condition and the level of partner disagreement (more regulation paired with less regulation, or more regulation paired with stay the same). Models run separately for each conversation length subgroup (0+ to 4+).

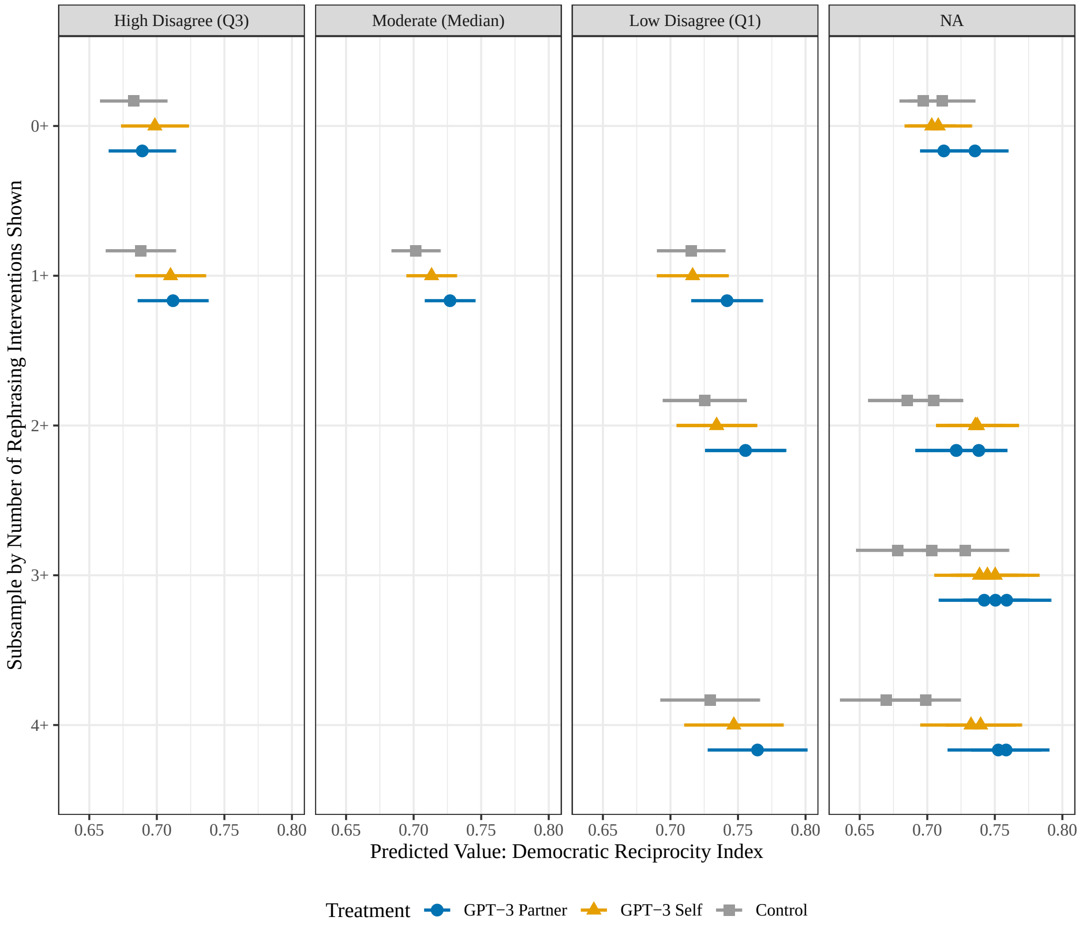

Figure S17: Predicted values of the Democratic Reciprocity Index value, based on an OLS model with an interaction between the treatment condition and the level of partner disagreement. Predicted values shown at quartile values of the difference in pre-survey gun policy attitude indices. Models run separately for each conversation length subgroup (0+ to 4+).

References

[1] Stephen Ansolabehere, Jonathan Rodden, and James M. Snyder. “The strength of issues: Using multiple measures to gauge preference stability, ideological constraint, and issue voting”. In: *American Political Science Review* 102.2 (2008), pp. 215–232.

[2] Adamantios Diamantopoulos et al. “Guidelines for choosing between multi-item and single-item scales for construct measurement: a predictive validity perspective”. In: *Journal of the Academy of Marketing Science* 40 (2012), pp. 434–449.

[3] Alan S Gerber and Donald P Green. *Field experiments: Design, analysis, and interpretation.* WW Norton, 2012.
